# Bioinformatic analysis of ESTs collected by Sanger and pyrosequencing methods for a keystone forest tree species: oak

**DOI:** 10.1186/1471-2164-11-650

**Published:** 2010-11-23

**Authors:** Saneyoshi Ueno, Grégoire Le Provost, Valérie Léger, Christophe Klopp, Céline Noirot, Jean-Marc Frigerio, Franck Salin, Jérôme Salse, Michael Abrouk, Florent Murat, Oliver Brendel, Jérémy Derory, Pierre Abadie, Patrick Léger, Cyril Cabane, Aurélien Barré, Antoine de Daruvar, Arnaud Couloux, Patrick Wincker, Marie-Pierre Reviron, Antoine Kremer, Christophe Plomion

**Affiliations:** 1 INRA, UMR 1202 BIOGECO, 69 route d'Arcachon, F-33612 Cestas, France; 2 Forestry and Forest Products Research Institute, Department of Forest Genetics, Tree Genetics Laboratory, 1 Matsunosato, Tsukuba, Ibaraki, 305-8687, Japan; 3 Plateforme bioinformatique Genotoul, UR875 Biométrie et Intelligence Artificielle, INRA, 31326 Castanet-Tolosan, France; 4 INRA/UBP UMR 1095, Laboratoire Génétique, Diversité et Ecophysiologie des Céréales, 234 avenue du Brézet, 63100 Clermont Ferrand, France; 5 INRA, UMR1137 EEF "Ecologie et Ecophysiologie Forestières", F 54280 Champenoux, France; 6 Université de Bordeaux, Centre de Bioinformatique de Bordeaux, Bordeaux, France; 7 CNRS, UMR 5800, Laboratoire Bordelais de Recherche en Informatique, Talence, France; 8 CEA, DSV, Genoscope, Centre National de Séquençage, 2 rue Gaston Crémieux CP5706 91057 Evry cedex, France

## Abstract

**Background:**

The Fagaceae family comprises about 1,000 woody species worldwide. About half belong to the *Quercus *family. These oaks are often a source of raw material for biomass wood and fiber. Pedunculate and sessile oaks, are among the most important deciduous forest tree species in Europe. Despite their ecological and economical importance, very few genomic resources have yet been generated for these species. Here, we describe the development of an EST catalogue that will support ecosystem genomics studies, where geneticists, ecophysiologists, molecular biologists and ecologists join their efforts for understanding, monitoring and predicting functional genetic diversity.

**Results:**

We generated 145,827 sequence reads from 20 cDNA libraries using the Sanger method. Unexploitable chromatograms and quality checking lead us to eliminate 19,941 sequences. Finally a total of 125,925 ESTs were retained from 111,361 cDNA clones. Pyrosequencing was also conducted for 14 libraries, generating 1,948,579 reads, from which 370,566 sequences (19.0%) were eliminated, resulting in 1,578,192 sequences. Following clustering and assembly using TGICL pipeline, 1,704,117 EST sequences collapsed into 69,154 tentative contigs and 153,517 singletons, providing 222,671 non-redundant sequences (including alternative transcripts). We also assembled the sequences using MIRA and PartiGene software and compared the three unigene sets. Gene ontology annotation was then assigned to 29,303 unigene elements. Blast search against the SWISS-PROT database revealed putative homologs for 32,810 (14.7%) unigene elements, but more extensive search with Pfam, Refseq_protein, Refseq_RNA and eight gene indices revealed homology for 67.4% of them. The EST catalogue was examined for putative homologs of candidate genes involved in bud phenology, cuticle formation, phenylpropanoids biosynthesis and cell wall formation. Our results suggest a good coverage of genes involved in these traits. Comparative orthologous sequences (COS) with other plant gene models were identified and allow to unravel the oak paleo-history. Simple sequence repeats (SSRs) and single nucleotide polymorphisms (SNPs) were searched, resulting in 52,834 SSRs and 36,411 SNPs. All of these are available through the Oak Contig Browser http://genotoul-contigbrowser.toulouse.inra.fr:9092/Quercus_robur/index.html.

**Conclusions:**

This genomic resource provides a unique tool to discover genes of interest, study the oak transcriptome, and develop new markers to investigate functional diversity in natural populations.

## Background

The distribution of adaptive genetic variation has become of upmost importance in domesticated and wild tree species for the management of natural resources and gene conservation [1]. Monitoring of genetic diversity for adaptive traits in plants is usually implemented in common garden experiments. Forest tree population geneticists have struggled for decades with the establishment of such experiments called provenance tests, aiming at exploring the range and distribution of genetic variation of fitness related traits. However, such investigations are extremely costly, as most traits can only be evaluated after trees have reached the adult stage. Hence provenance and progeny tests have been mainly limited to woody species of short rotation and of economic importance, and usually undergoing intensive breeding efforts. Species of lower economic interests or long generation tree species, for which no breeding activities could be conducted, have been largely unexplored for their natural genetic variation. For these species, genomics may offer a short cut to field tests for exploring gene diversity, provided that genes of adaptive significance have been identified. In this respect, our objective was to develop an extensive catalogue of gene sequences that can be used for exploring genetic variation in natural populations of tree species of widespread ecological importance such as oaks.

Oaks belong to the genus *Quercus*, which comprises several hundred diploid and highly heterozygous species spreading throughout the northern hemisphere, from the tropical to the boreal regions [2]. The distribution encompasses strong ecological and climatic gradients in the Eurasiatic as in the American continents, in an almost continuous pattern. Throughout its natural range, the genus has differentiated into numerous species and populations adapted to extremely variable habitats from swamps to deserts and from sea level up to 4,000 meters in the Himalayas, and expressed in very different life history traits [3]. Because of their longevity and their very large geographic distribution, oaks are also key drivers of terrestrial biodiversity as they harbour large communities of insects, fungi, and vertebrates [4]. We expect that the discovery of genes of adaptive significance in oaks may therefore lead to significant progress in the evolutionary genetics and ecology of whole communities.

The genus *Quercus *belongs to the Fagaceae family, that comprises other genera of ecological significance mainly *Castanea *(chestnuts), *Fagus *(beeches), *Castanopsis *and *Lithocarpus *[5]. Phylogenetic and historical investigations suggest a very rapid differentiation among the different genera, and the persistence of strong genetic relationships, especially between *Quercus, Castanea, Castanopsis*, and *Lithocarpus *[6,7]. Indeed earlier reports of comparative mapping indicate strong macrocolineratity within linkage groups between *Quercus *and *Castanea *[8-10]. Hence, gene sequences of oaks may lead to a broad range of genetic investigations within one of the largest distributed tree family.

In this study we describe the construction and analyse the content of wide EST data corresponding to the first large scale exploration of the transcriptome of oaks. A total of 34 cDNA libraries were constructed from mRNA extracted from bud, leaf, root and wood forming tissue of the two main European white oak species (*Quercus petraea*, sessile oak and *Q. robur*, pedunculate oak). Tissues were also collected from abiotically challenged trees. After clustering and contig assembly of EST sequences, comparative analysis was conducted to assign functional annotation through similarity searches. The project led to the construction of a biological resource accessible at the repository centre established by the Evoltree network of excellence http://www.evoltree.eu/index.php/repository-centre and a database where assembly and annotations and putative SNP and SSR markers are available http://genotoul-contigbrowser.toulouse.inra.fr:9092/Quercus_robur/index.html. This resource is vitally important not only for genomic and genetic research in oaks and related species, but also for larger communities harboured by oak ecosystems.

## Results and Discussion

**Figure 1:**
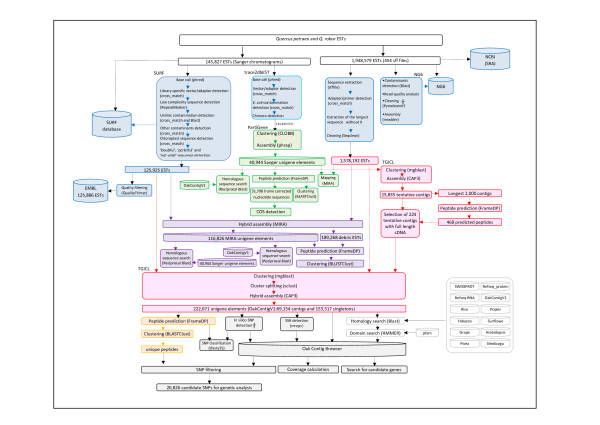
**Schematic representation of the bioinformatic analysis**. Sequence processing, storage, assembly, annotation and SNP/SSR detection. The † mark indicates logical link between duplicated reads and SNP detection.

A schematic representation of data processing is shown in Figure 1.

### Sanger sequencing

We produced 145,827 Sanger reads from 20 cDNA libraries (Tables 1 and 2), including ten libraries from *Q. petraea *and ten from *Q. robur*. There were nine Suppression Subtractive Hybridization (SSH) and 11 cDNA libraries. We used five different experimental systems (kit combinations) for library construction. Two systems were used for SSH libraries, while three systems were used for cDNA libraries. The maximum and minimum number of genotypes in a library was 60 for library D and 2 for libraries B and I, respectively. There were seven bud, seven root, four leaf and two differentiating secondary xylem libraries. All sequences were subjected to pre-processing (SURF and qualityTrimmer software, see Methods section) to remove library specific cloning-vector and adaptor sequences, to mask low complexity sequences, to eliminate contaminants and poor-quality sequences (e.g. very short reads). The resulting Sanger catalogue contained 125,886 high quality sequences that are available at the EST section of EMBL. Furthermore, all of the chromatograms can be downloaded from the SURF web site http://surf.toulouse.inra.fr/ with user name (oak) and password (oak1).

**Table 1:** Oak (Q. petraea and Q. robur) cDNA libraries for Sanger sequencing

Species	Library code	Library name	Library type	Kit for library construction	No. of genotypes	Tissue	Sample stage/treatment
*Q. petraea*	A	LG0BAC	Standard	LambdaZAP	50	bud	Quiescent buds from 2-year-old trees (Phalsbourg (57-F) and Mirecourt (88-F)) sampled in April 7^th ^and 9^th^, 2004
	B	QpBudslate	Standard	CloneMiner	2	bud	Early swelling bud sampled in March 24^th ^and 30^th ^, 2006 on adult trees
	C	QpSwellingBud	Subtractive	SMART PCR, BD PCR-Select, pCR4 TOPO	20	bud	Swelling vs. quiescent buds, 1-year-old trees
	D	QpBudquiescent	Subtractive	SMART PCR, BD PCR-Select, pCR4 TOPO	60	bud	Developing (internodes have started to grow) vs. quiescent buds, 1-year-old trees
	E	QpVegetativeGrowth	Subtractive	SMART PCR, BD PCR-Select, pCR4 TOPO	20	bud	Quiescent vs. swelling buds, 1-year-old trees
	F	sessile48hours	Subtractive	SMART PCR, BD PCR-Select, pGEM-T easy	10	root	Hypoxia for 24 and 48 h. White roots from 6-month-old cuttings, 2005
	G	sessile6 hours	Subtractive	SMART PCR, BD PCR-Select, pGEM-T easy	10	root	Hypoxia for 6 h. White roots from 6-month-old cuttings, 2005
	H	Qp5stressRoots	Standard	CloneMiner	15	root	6-month-old seedlings collected in October 2006, 1/10°C 3 days, 2/35°C 4 days, 3/CO_2 _700 ppm, 4/water stress, 5/hypoxie 48 h
	I	QpLeaf5stress	Standard	CloneMiner	15	leaf	6-month-old seedlings collected in October 2006 : 1/10°C 3 days, 2/35°C 4 days, 3/CO_2 _700 ppm, 4/water stress, 5/hypoxie 48 h
	J	QpXyleme	Standard	Creator SMART	2	xylem	Secondary differentiating xylem sampled in May 21^th^, 2007 on adult trees

*Q. robur*	K	QrBudsEarly	Standard	CloneMiner	3	bud	Setting bud sampled in October 26^th^, 2006 on adult trees
	L	QrBudslate	Standard	CloneMiner	3	bud	Swelling buds sampled in March 24^th ^and 30^th^, 2007 on adult trees
	M	LG00BAD	Standard	LambdaZAP	10	root	Fine roots under optimal fertilization and Irrigation conditions, harvested in August 2004 [48]
	N	pedonculate6 hours	Subtractive	SMART PCR, BD PCR-Select, pGEM-T easy	10	root	Hypoxia for 6 h. White roots from 6-month-old cuttings, 2005
	O	pedonculate48 hours	Subtractive	SMART PCR, BD PCR-Select, pGEM-T easy	10	root	Hypoxia for 24 and 48 h. White roots from 6-month-old cuttings, 2005
	P	LG0BAA	Standard	LambdaZAP	3	leaf	Young leaves sampled on adult trees in April 27^th^, 2004
	Q	HighWUE	Subtractive	SMART PCR, BD PCR-Select, pGEM-T easy	5	leaf	Green leaves on one-year-old cuttings, (high vs. low WUE) October 2005
	R	LowWUE	Subtractive	SMART PCR, BD PCR-Select, pGEM-T easy	5	leaf	Green leaves on one-year-old cuttings, (low vs. high WUE) October 2005
	S	LG0BAB	Standard	LambdaZAP	3	xylem	Secondary differentiating xylem sampled on adult trees in April 27^th^, 2004
	T	QrAnoxie	Standard	Creator SMART	10	root	Hypoxia for 24 and 48 h. White roots from 6-month-old cuttings, 2005

**Table 2:** Sequencing statistics for libraries sequenced by the Sanger method

Library code	No. reads (I)	No of 3' reads in (1)	No. reads in OakContigV1 assembly	Number of high quality sequences (2)	Sequencing success rate % (2)/(1)	Average length (bp) in (2)
A	10717	0	10317	10313	96.3%	535
B	9615	4711	6673	6669	69.4%	517
C	392	0	224	224	57.1%	313
D	184	0	110	110	59.8%	377
E	203	0	148	148	72.9%	404
F	2493	0	2305	2305	92.5%	566
G	1756	0	1604	1604	91.3%	500
H	18935	4747	12002	11989	63.4%	505
I	19195	4868	15424	15424	80.4%	597
J	9377	4491	8964	8964	95.6%	589
K	9578	0	8652	8649	90.3%	620
L	9500	0	8533	8525	89.8%	575
M	19685	513	18756	18753	95.3%	583
N	1700	0	1518	1518	89.3%	509
O	2129	0	1975	1975	92.8%	534
P	7513	0	7238	7237	96.3%	712
Q	1765	0	1589	1589	90.0%	522
R	1768	0	1507	1507	85.2%	495
S	10164	0	9951	9950	97.9%	584
T	9158	4279	8435	8433	92.1%	604

Total	145827	23609	125925	125886	86.4%	575

Library codes are as in Table 1.

The sequencing success rate (defined as the number of high quality reads divided by the total number of sequences) as well as the average length of high quality sequences varied depending on libraries (Table 2). Excluding the three Suppression Subtractive Hybridization (SSH) libraries (C, D, E) for which these two parameters were among the lowest, the former parameter ranged from 69% to 97.9% and the latter from 500 bp to 712 bp. Overall, the average read length of high quality sequences was 575 bp.

SURF detected 402 chloroplastic (cp) reads, corresponding to a global rate of 0.28% (Table 3). cp sequences were detected in all tissue types, including leaves and buds at quite high rate, ranging from 2 to 10% (libraries C, D, Q, R), but also in root (M) and xylem (J) at a much lower rate. There were three reads that matched with *E. coli *sequence. SURF tagged a total of 22,431 sequences as 'not valid' (short and/or contaminant sequences) including 1,555 and 229 sequences flagged as 'doubtful' (containing library specific short vector/adaptor sequences), and 'pcrkitful' (containing short sequences from the UniVec database), respectively. Interestingly, most of these low quality sequences were found in libraries constructed with the lambdaZap kit (A, M, P, S) as well as in three SSH libraries (C, D, E). It should also be noted that about 1/3 (544) of the 'doubtful' sequences could be attributed without ambiguity to chimeras, because they presented library specific sequences in the [30%-70%] interval of the sequence. Trace2dbEST pipeline [11] also detected 63 reads with putative chimeras. Out of 63 chimeras judged by this pipeline, only one read was common with those detected by SURF. The difference may result from the different phred [12,13] and cross_match [14] parameters. Trace2dbEST uses phred error probability cut-off of 0.05 (which corresponds to quality value (QV) of 13), while that in SURF was 0.01 (QV = 20). The sequence regions with the specified QV were expected to be longer in sequences processed by trace2dbEST than by SURF. Theoretically, the probability to detect a chimera is higher for larger sequences. Unfortunately, trace2dbEST had less stringent parameters in cross_match compared to SURF, which decreased the rate of chimera detection. This comparison clearly shows that parameter optimization to detect possible chimera is necessary for each study objectives. For example, if the goal is to provide a global view of the transcriptome, chimeric clones do not cause large problems. However, if the goal is to bioinformatically infer full-length cDNAs, chimeric clones must be strictly eliminated.

**Table 3:** SURF process summary

Library code	No. of reads (a)	No. match with chloroplast (b)	% match with chloroplast (b)/(a)	No. doubtful sequences (c)	% doubtful (c)/(a)	No. PCRkitful sequences (d)	% of PCRkit (d)/(a)	No. 'not valid'	% of 'Not valid'
A	10717	20	0.19%	163	1.52%	19	0.18%	647	6.04%
B	9615	1	0.01%	14	0.15%	7	0.07%	2951	30.69%
C	392	21	5.36%	7	1.79%	26	6.63%	204	52.04%
D	184	19	10.33%	1	0.54%	31	16.85%	100	54.35%
E	203	0	0.00%	14	6.90%	34	16.75%	76	37.44%
F	2493	7	0.28%	3	0.12%	5	0.20%	207	8.30%
G	1756	5	0.28%	0	0.00%	5	0.28%	164	9.34%
H	18935	5	0.03%	25	0.13%	1	0.01%	6976	36.84%
I	19195	4	0.02%	28	0.15%	5	0.03%	3815	19.87%
J	9377	64	0.68%	15	0.16%	4	0.04%	518	5.52%
K	9578	3	0.03%	24	0.25%	10	0.10%	955	9.97%
L	9500	2	0.02%	19	0.20%	3	0.03%	989	10.41%
M	19685	48	0.24%	862	4.38%	3	0.02%	1894	9.62%
N	1700	7	0.41%	0	0.00%	9	0.53%	191	11.24%
O	2129	4	0.19%	1	0.05%	3	0.14%	173	8.13%
P	7513	12	0.16%	194	2.58%	28	0.37%	492	6.55%
Q	1765	84	4.76%	1	0.06%	12	0.68%	265	15.01%
R	1768	37	2.09%	2	0.11%	4	0.23%	306	17.31%
S	10164	5	0.05%	160	1.57%	19	0.19%	688	6.77%
T	9158	54	0.59%	22	0.24%	1	0.01%	820	8.95%

Total	145827	402	0.28%	1555	1.07%	229	0.16%	22431	15.38%

Library codes are as in Table 1.

### 454-sequencing

We constructed nine libraries from *Q. petraea *and five from *Q. robur *(Tables 4 and 5). Six libraries were established from mRNA extracted from the parental lines of mapping pedigrees and consisted of leaves and buds from single individuals. They were sequenced by 454 Titanium. There were four libraries from buds, two from leaves and buds and two from flowers. These eight libraries were sequenced by 454 GS-FLX. In total 1,948,579 reads were produced. The average read length varied from 167 to 211 bp for libraries sequenced by GS-FLX and from 350 to 390 bp for those sequenced by Titanium (Table 5). We used the NG6 pipeline (see Methods section) to detect contaminants by Blast search and found yeast sequences in nine out of 14 libraries (additional file 1: TableS1). No *E. coli *and phage sequences were found in libraries sequenced by GS-FLX, while they were detected in all the libraries sequenced by Titanium. Average depth estimated based on the Newbler assembler was higher (varying from 7.32 to 10.08) and unique sequence rate was lower (varying from 16.9% to 23.9%) for libraries sequenced by Titanium compared to GS-FLX (additional file 2: TableS2).

**Table 4:** Oak (Q. petraea and Q. robur) cDNA libraries for 454-pyrosequencing

Species	Library code	Library name	Library type	Kit for library construction	No. of genotypes	Tissue	Sample stage/treatment
*Q. petraea*	I	LC1-EcoEndoDorm	Standard	SMART PCR cDNA Synthesis Kit	30	Buds	Endodormancy, sampled in September 17^th ^and 24^th ^and October 1^st^, 2005
*Q. petraea*	II	LC2-EcoEndoDorm	Standard	SMART PCR cDNA Synthesis Kit	30	Buds	Ecodormancy, sampled in January 14^th ^and 28^th ^and February 11^th^, 2005
*Q. petraea*	III	SJ1-EcoEndoDorm	Standard	SMART PCR cDNA Synthesis Kit	30	Buds	Endodormancy, sampled in September 17^th ^and 24^th ^and October 1^st^, 2005
*Q. petraea*	IV	SJ2-EcoEndoDorm	Standard	SMART PCR cDNA Synthesis Kit	30	Buds	Ecodormancy, sampled in January 14^th ^and 28^th ^and February 11^th^, 2005
*Q. petraea*	V	10QS-Intersp	Standard	SMART PCR cDNA Synthesis Kit	10	Leaves, buds	Young and mature leaves, quiescent and later buds
*Q. robur*	VI	10QP-intersp	Standard	SMART PCR cDNA Synthesis Kit	10	Leaves, buds	Young and mature leaves, quiescent and later buds
*Q. petraea*	VII	FS	Standard	SMART PCR cDNA Synthesis Kit	2	Flower	Pollen, flowers
*Q. robur*	VIII	FP	Standard	SMART PCR cDNA Synthesis Kit	2	Flower	Pollen, flowers
*Q. petraea*	IX	Qs21	Normalized	MINT cDNA synthtesis Kit,	1	Leaves, buds	Quiescent, swelling buds; young, mature leaves
*Q. petraea*	X	Qs28	Normalized	MINT cDNA synthtesis Kit,	1	Leaves, buds	Quiescent, swelling buds; young, mature leaves
*Q. petraea*	XI	Qs29	Normalized	MINT cDNA synthtesis Kit,	1	Leaves, buds	Quiescent, swelling buds; young, mature leaves
*Q. robur*	XII	3P	Normalized	MINT cDNA synthtesis Kit,	1	Leaves, buds	Quiescent, swelling buds; young, mature leaves
*Q. robur*	XIII	11P	Normalized	MINT cDNA synthtesis Kit,	1	Leaves, buds	Quiescent, swelling buds; young, mature leaves
*Q. robur*	XIV	A04	Normalized	MINT cDNA synthtesis Kit,	1	Leaves, buds	Quiescent, swelling buds; young, mature leaves

**Table 5:** Sequence statistics for libraries sequenced by 454-pyrosequencing

Library name	454	Number of reads (3)	Average length (bp) in (3)	Number of reads in OakContigV1
LC1-EcoEndoDorm	GS-FLX	115050	167	70019
LC2-EcoEndoDorm	GS-FLX	137380	179	98725
SJ1-EcoEndoDorm	GS-FLX	79345	183	44732
SJ2-EcoEndoDorm	GS-FLX	164140	203	138921
10QS-Intersp	GS-FLX	159478	211	131932
10QP-Intersp	GS-FLX	99472	205	80748
FS	GS-FLX	112207	194	86838
FP	GS-FLX	154819	196	117518
QS21	Titanium	153558	374	132870
QS28	Titanium	124143	390	110304
QS29	Titanium	206828	386	182675
11P	Titanium	137409	381	119869
3P	Titanium	143969	387	127339
A04	Titanium	160781	350	135523

Total		1948579	281	1578013

**Figure 2:**
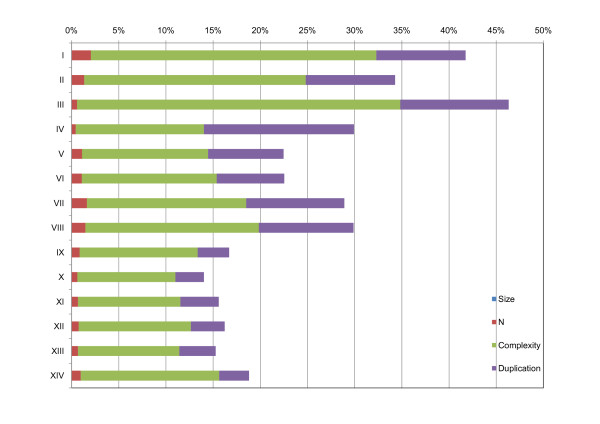
**Results of pyrocleaner on 454-reads**. Size: sequences with more than or less than two standard deviation from the mean length; N: sequences with more than 4% of N call; Complexity: low complexity sequences; Duplication: possible PCR artefacts during emulsion PCR. The portion of reads in the size criteria is too small (0.002% - 0.025%) to be seen.

We used the pyrocleaner program from NG6 to identify too short or too long sequences, dirty sequences, low complexity sequences and duplicated reads as defined in the Methods section. Depending on the library, from 14.0% (library X) to 46.3% (library III) of the reads presented these features (Figure 2). Overall, libraries sequenced by Titanium showed lower number of low quality sequences (from 14.0% in library X to 18.8% in library XIV), while those sequenced by GS-FLX showed higher values (from 22.5% in library V to 46.3% in library III). In particular, the duplication rate was higher in libraries sequenced by GS-FLX (7.2% to 15.9%) compared to Titanium (3.0% to 4.1%).

### Coverage of transcripts within libraries

**Figure 3:**
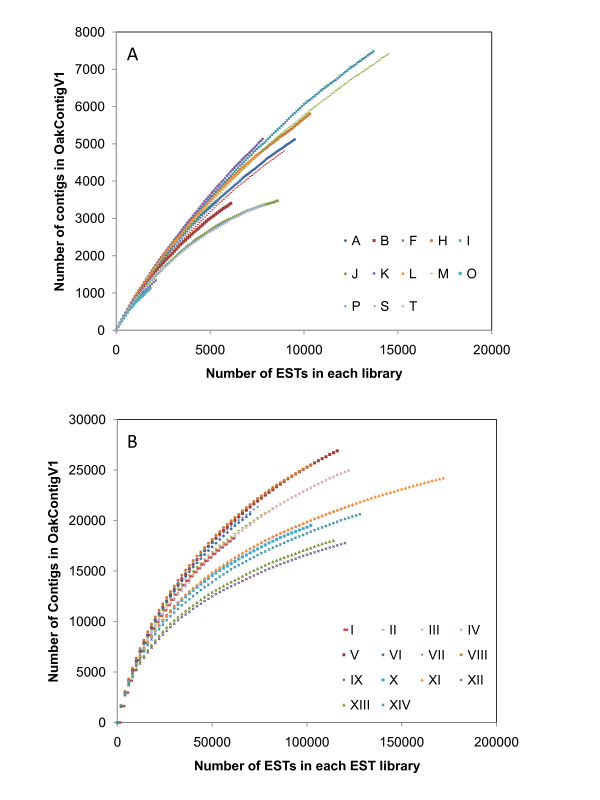
**Relationship between number of ESTs in a library and the number of contigs in OakContigV1**. The library codes are as in Table 1 for (A) Sanger (from A to T) and Table 4 for (B) 454 pyrosequencing (from I to XIV).

Transcript coverage was estimated by inferring relationship between number of ESTs in a library and number of contigs (Figure 3) as detailed in the Methods section. For libraries sequenced by the Sanger method (Table 6), the maximum coverage (82.7%) was obtained for library J, followed by library T (80.7%). These two libraries were constructed by Creator Smart Kit (Table 1) and sequenced from both 3' and 5' directions (Table 2). They showed the plateau for the number of contigs in OakContigV1 at the number of ESTs around 8,000 (Figure 3A). This means 8,000 ESTs were enough to represent the transcripts in these libraries. On the other hand, library K showed the minimum transcript coverage of 48.9% with 8,652 ESTs (Table 2). To achieve the coverage greater than 80% in library K, about 18,000 ESTs may have been necessary. Libraries sequenced by pyrosequencing achieved a coverage greater than 80% (Table 6). Furthermore, for libraries sequenced by Titanium platform (libraries from IX to XIV), the coverage was virtually 100%, which was probably attained by both mixing RNAs from different tissues and normalization procedure used to construct these libraries (Table 4).

**Table 6:** Coverage analysis for each library

**Sequencing method**^**#**^	Library code	A	B	Number of contigs in OakContigV1 (C)	Coverage (%) C/A
S	A	7550.3	-1.17E-04	5122	67.8%
S	B	5186.4	-1.73E-04	3420	65.9%
S	F	2515.9	-3.59E-04	1353	53.8%
S	H	8802.1	-1.02E-04	5809	66.0%
S	I	12232.2	-6.82E-05	7485	61.2%
S	J	4218.9	-2.03E-04	3487	82.7%
S	K	10541.7	-8.48E-05	5158	48.9%
S	L	8623.1	-1.04E-04	4704	54.6%
S	M	11485.6	-7.02E-05	7409	64.5%
S	O	1920.3	-4.77E-04	1141	59.4%
S	P	7184.0	-1.22E-04	4005	55.7%
S	S	7542.7	-1.11E-04	4816	63.8%
S	T	4194.4	-2.02E-04	3385	80.7%

P	I	20631.3	-3.29E-05	18245	88.4%
P	II	23225.8	-2.69E-05	21314	91.8%
P	III	16931.9	-4.31E-05	14169	83.7%
P	IV	26459.2	-2.03E-05	25038	94.6%
P	V	29306.1	-1.94E-05	27081	92.4%
P	VI	23674.9	-2.72E-05	20702	87.4%
P	VII	23941.0	-2.78E-05	21381	89.3%
P	VIII	27733.7	-2.22E-05	25588	92.3%
P	IX	21725.9	-2.42E-05	21620	99.5%
P	X	19916.9	-2.80E-05	19645	98.6%
P	XI	23860.4	-1.98E-05	24265	101.7%
P	XII	17736.9	-2.61E-05	17830	100.5%
P	XIII	18021.2	-2.71E-05	18065	100.2%
P	XIV	20576.5	-2.41E-05	20616	100.2%

Library codes are as in Tables 1 and 4.

^#^S: Sanger method; P: pyrosequencing method

### Assembly

We produced three kinds of assemblies using three different approaches (Table 7). First, we processed the 134,500 Sanger reads (Table 8) resulting from the trace2dbEST analysis using the PartiGene pipeline[15] and produced 40,944 unigene elements, containing 17,499 contigs and 23,445 singletons (Table 7). Contigs were defined in sequence assembly, resulting from multiple reads, while singletons were unique sequence that were not clustered with any other reads or that were not assembled with any other reads in a cluster. The distribution of sequence length (bp) of contigs and singletons is indicated in additional file 3: Figure S1 (A). While the mode of the distribution resided in the (600-700] class for both singletons and contigs, the average and maximum length of singletons and contigs were quite different: 919 bp and 4,412 bp, respectively for the contigs, and 485 bp and 1,305 bp for the singletons. Of the 17,499 contigs, 6,271 (35.8%) contained two ESTs, 3,104 (17.7%) contained three ESTs, 1,842 (10.5%) contained four ESTs, 1,257 (7.2%) contained five ESTs and 5,025 (28.7%) contained more than five ESTs (additional file 4: Figure S2, red bars). The average and maximum number of ESTs in a single contig was 6.3 and 510, respectively. The average GC content of this unigene sets was 41.6%. When we compared these results with other similar studies (Table 9), the statistics of the oak assembly were within the range of what has been reported so far in plants. Positive relationships between the different quantities (numbers of ESTs, unigene elements, contigs, and singletons) were evident. Even though the number of ESTs collected was different for each study, the percentages of contigs within each unigene set was nearly identical (mean = 41.1%), except for the cocoa EST assembly.

**Table 7:** Statistics for assembly by PartiGene (Sanger ESTs only), MIRA and TGICL (Sanger and 454- ESTs)

	PartiGene	MIRA	TGICL (OakContigV1)
Number of Sanger/454 reads included in assembly	134500/0	125925/1578013	125925/1578013
Number of contigs (average length (bp))	17499 (919)	113625 (671)	69154 (705)
Number of singletons (average length (bp))	23445 (485)	3201^# ^(236)	153517 (300)
Number of unigene elements (contigs + singletons)	40944	116826	222671
Number of reads in contigs	108626	1511639	1550824

# Debris was excluded. MIRA classified 189,268 reads as debris. If debris is considered as singletons, the number of singletons and unigenes elements by MIRA would become 192,469 and 306,094, respectively.

**Table 8:** PartiGene assembly summary for libraries sequenced by the Sanger method

Library code	No. reads assembled (a)	No. contigs (b)	No. singletons (c)	No. unigene elements (b+c)	Redundancy (b+c)/(a)
A	10515	1858	3885	5743	54.62%
B	8522	1610	3276	4886	57.33%
C	242	43	34	77	31.82%
D	116	20	11	31	26.72%
E	152	34	61	95	62.50%
F	2353	373	1128	1501	63.79%
G	1628	240	902	1142	70.15%
H	14612	2585	5725	8310	56.87%
I	17166	2451	7115	9566	55.73%
J	9046	2478	927	3405	37.64%
K	9004	1234	4594	5828	64.73%
L	9018	1247	4466	5713	63.35%
M	19319	2883	7606	10489	54.29%
N	1558	223	918	1141	73.23%
O	2021	314	964	1278	63.24%
P	7373	1227	3031	4258	57.75%
Q	1618	251	813	1064	65.76%
R	1552	211	821	1032	66.49%
S	10058	1569	4094	5663	56.30%
T	8627	2208	1101	3309	38.36%

Total	134500	17499	23445	40944	30.44%

Library codes are as in Table 1.

**Table 9:** Comparison of EST sequencing statistics for Sanger sequencing

Organisms	Number of ESTs (a)	Contigs (b)	Singletons (c)	Number of Unigenes (b + c)	% of contig (b/(b + c))	Redundancy ((b + c)/a)	References
Oak	134500	17499	23445	40944	42.7%	30.4%	This study
Cotton	153969	22030	29077	51107	43.1%	33.2%	Udall et al. [66]
Cocoa	149650	12692	35902	48594	26.1%	32.5%	Argout et al. [67]
Spruce	147146	19941	26804	46745	42.7%	31.8%	Ralph et al. [68]
Actinidia	132577	18070	23788	41858	43.2%	31.6%	Crowhurst et al. [69]
Poplar	102019	15574	19563	35137	44.3%	34.4%	Sterky et al. [70]
Lotus	74472	8503	11954	20457	41.6%	27.5%	Asamizu et al. [71]
Citrus	52626	7120	8544	15664	45.5%	29.8%	Terol et al. [72]

**Figure 4:**
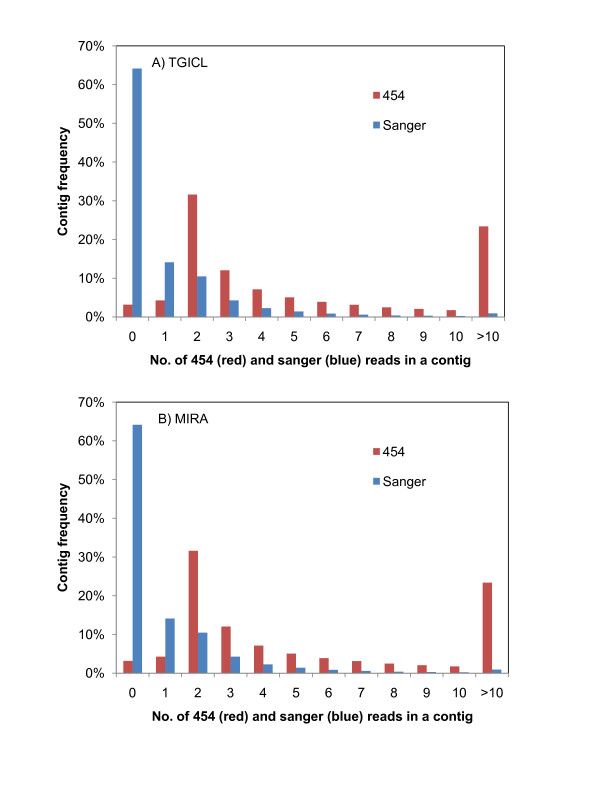
**Composition of contigs constructed by (A) TGICL (OakContigV1) and (B) MIRA software**. When the number of Sanger reads is zero in a contig, it means that the contig is made up of only 454-reads (the blue bar at zero on the horizontal axis). On the other hand, when the number of 454-reads is zero in a contig, it means that the contig is made up of only Sanger reads (the red bar at zero on the horizontal axis).

Second, we used the SIGENAE system (which relies on the TGICL software [16], see Methods section) to bring together in the same analysis 125,925 Sanger and 1,578,192 454-reads. Overall, 222,671 elements (69,154 (31%) tentative consensus sequences (TCs) and 153,517 (69%) singletons; OakContigV1) were obtained (Table 7). The average and maximum length of contigs was 705 bp and 7,898 bp, respectively. The distribution of sequence length (bp) of contigs and singletons is indicated in additional file 3: Figure S1 (B). The distribution was bimodal for both contigs and singletons. The first peak for singletons resided in (200-300] class, where 56,249 GS-FLX reads (92.5% of total reads within the class) resided. The second peak for singletons laid in the (400-500] class, where 14,336 Titanium reads (95.7% of total reads within the class) resided. For contigs, the mode was located at the (200-300] class. Within this class, there were 11,269 contigs (92.0% of total contigs within the class) that were made up from GS-FLX reads only. The average and maximum depth of contigs was 22.4 and 4,927, respectively. The deepest contig was 1,336 bp and presented similarity with a chloroplast membrane protein from *Mercurialis perennis *at e-value of 6e-10. Of the 69,154 TCs, 23,281 (33.7%) contained two ESTs, 8,860 (12.8%) contained three ESTs, 5,069 (7.3%) contained four ESTs and 31,944 (46.2%) contained more than four ESTs (additional file 4: Figure S2, green bars). Overall the 69,154 TCs contained 1,550,824 sequences. Among the 69,154 TCs, 40,542 (58.6%) consisted of 454-reads only, while 1,230 (1.78%) were made up of Sanger reads only (Figure 4A). In total, 356,893 (22.6%) of the 454-reads did not cluster to Sanger reads (139,443 singletons plus 217,450 454-reads in 40,542 TCs supported only by 454-reads). This also means that 77.4% of the 454-reads clustered with Sanger reads. The average GC content of OakContigV1 was 39.8%.

Graphical interface to browse OakContigV1 was constructed using the Ensembl tool (oak contig browser; http://genotoul-contigbrowser.toulouse.inra.fr:9092/Quercus_robur/index.html (user: oak, pass word: quercus33)). Browsing similarity annotation, SNP alignments and data mining by BioMart are also available as described in detail in Fleury et al. [17]. All of the data can be downloaded from the web site.

Third, we used MIRA software [18] to produce direct 454-Sanger hybrid assembly. This analysis resulted into 116,826 unigene elements including 113,625 contigs and 3,201 singletons (Table 7). There were also 189,268 so called "debris" reads, including 12,532 Sanger and 176,736 454-reads. About 54.6% (103,428 reads out of 189,268) of the sequences in the 'debris' corresponded to 67.4% of the OakContigV1 singletons. The number of Sanger and 454-reads included in assembly was 125,925 and 1,578,013, respectively. The distribution of sequence length (bp) of contigs and singletons is indicated in additional file 3: Figure S1 (C). For contigs, the mode was located on the (200-300] class. Within this class, there were 16,601 contigs (86.6% of total contigs within the class) that were made up of GS-FLX reads only. Among the 113,625 contigs, 72,896 (64.2%) consisted of 454-reads only, while 3,582 (3.2%) were made up of Sanger reads only (Figure 4B). In total, 421,391 (26.7%) of the 454-reads did not cluster to Sanger reads (2,992 singletons plus 418,399 454-reads in 72,896 TCs supported by 454-reads only). This also means that 74.3% of the 454-reads clustered together with Sanger reads, a similar (although larger) value to that obtained using TGICL. The average and maximum length of contigs was 671 bp and 15,177 bp, respectively (additional file 3: Figure S1 (C)). The average and maximum depth of contigs was 13.3 and 3,253, respectively, that is almost twice smaller than that obtained with TGICL. Of the 113,625 TCs, 38,730 (34.1%) contained two ESTs, 15,189 (13.4%) contained three ESTs, 8,686 (7.6%) contained four ESTs and 51,020 (44.9%) contained more than four ESTs. Overall the 113,625 TCs contained 1,511,639 sequences. The average GC content of this unigene sets was 41.9%. A total of 33.5% of the reads that did not clustered with Sanger reads using either MIRA or TGICL assembler were identical.

Reciprocal best Blast hits (RBHs) were searched between unigene elements constructed by MIRA and TGICL, PartiGene and TGICL, as well as between PartiGene and MIRA. In total 32,459 sequences were identified as RBH between MIRA and TGICL (OakContigV1) unigene elements, which accounted for 27.8% and 14.6% of MIRA and OakContigV1 unigene elements, respectively. In terms of contigs, 27.9% of MIRA and 38.1% OakContigV1 contigs had RBHs, while in terms of singletons, 24.0% of MIRA and 4.0% of OakContigV1 singletons presented RBHs. This low percentage is due to the fact that MIRA classified most of the singletons as 'debris'. There were 17,933 RBHs between PartiGene and OakContigV1 unigene elements, which accounted for 43.8% and 8.05% of PartiGene and OakContigV1 unigene elements, respectively. In terms of contigs, 59.8% of PartiGene and 19.6% OakContigV1 contigs had RBHs, while in terms of singletons, 32.2% of PartiGene and 2.83% of OakContigV1 singletons had RBHs. There were 13,037 RBHs between PartiGene and MIRA unigene elements, which accounted for 31.8% and 11.2% of PartiGene and MIRA unigene elements, respectively. In terms of contigs, 51.4% of PartiGene and 11.4% of MIRA contigs had RBH, while in terms of singletons, 17.2% of PartiGene and 1.1% of MIRA singletons presented RBHs.

By the addition of 454-reads, the number of unigene elements was greatly increased from 40,944 based on the PartiGene assembly to 222,671 in OakContigV1. This is due to 139,443 454-singletons and 40,542 contigs that contain only 454-reads. In total, these 179,985 454-unigene elements accounted for 80.8% of the OakContigV1 sequences, comprising 22.6% of the 454-reads. It should also be pointed out that 46.8% (i.e. 10,073 Sanger reads) of the 21,504 PartiGene singletons also present in OakContigV1 were present as contig member of the TGICL assembly. In addition, mapping 454-reads onto the PartiGene assembly (using MIRA) showed that 852,986 (54.0%) reads were mapped, including 683,768 (43.3%) reads on contigs. The rest of the 454-reads did not find corresponding sequences within the PartiGene Sanger assembly. Because 77.4% of 454-reads were assembled with at least one Sanger read in OakContigV1, this simple mapping procedure resulted into a much lower rate of integration of 454-reads. All together, these results indicate the value of the combined assembly approach based on TGICL and the added value of 454-reads to assemble Sanger reads into contigs. When the assembly was carried out based on 454-reads only using the MIRA assembler, we found that 2698 (2.3%) decrease in the number of unigene elements, 60.7 bp (9.0%) decrease in the length of contigs. Sanger reads contributed more to the length of contigs than to the number of unigene elements.

### Detection of unique peptide elements

Starting from 222,671 unigene elements in OakContigV1, FrameDP [19] predicted peptides for 117,311 (52.7%) of them (additional file 5: Figure S3), resulting in 132,406 predicted peptides. A single peptide was predicted for 104,172 (46.8%) elements of OakContigV1, while the rest produced multiple peptides. The maximum number of predicted peptides from one sequence of OakContigV1 was seven. When 116,826 unigene elements plus the 189,268 'debris' produced by MIRA were used for peptide prediction ('debris' were included here for comparative purpose with TGICL analysis), FrameDP predicted peptides for 176,324 (57.6%) elements. For 164,468 (53.7%) of them, there was only one peptide predicted by FrameDP. When peptide prediction was performed for the unigene elements produced by PartiGene, 31,798 (77.7%) presented at least one peptide. Only one peptide was predicted for 27,273 (66.6%) unigene elements. Therefore, unigene elements of OakContigV1 presented the largest portion of non-translated sequences (additional file 5: Figure S3). Unigene elements from MIRA analysis also presented a large portion of non-translated sequence, due to 'debris' reads. When the 'debris' were excluded for peptide prediction, both MIRA and PartiGene displayed similar patterns of distribution of predicted peptides (data not shown). Only 41.3% singletons of OakContigV1 and 28.2% of 'debris' in MIRA, respectively, had at least one predicted peptide, while 67.6% of the singletons in PartiGene presented at least one predicted protein. Focusing on the contigs, 91.2%, 77.4% and 77.7% of PartiGene, OakContigV1 and MIRA elements, respectively, had at least one predicted peptide. Of 132,406 predicted peptides from OakContigV1, 91,148 (68.8%) had N-terminal or C-terminal peptide, while the rest (31.2%, i.e. 41,310 elements) was assumed to be full-length peptide with both start and stop codons identified.

**Figure 5:**
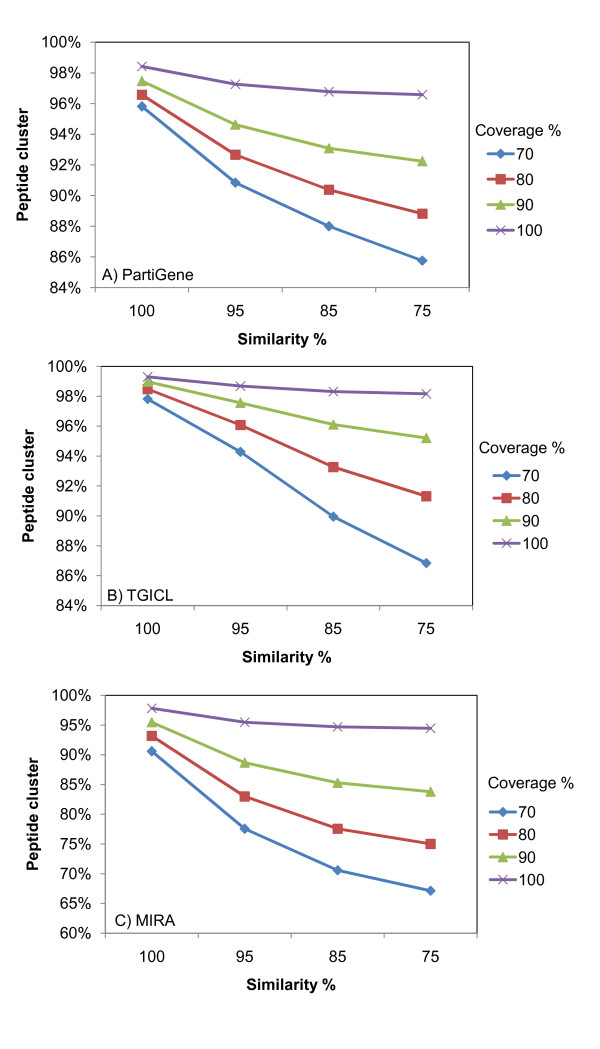
**BLASTClust clustering of peptides predicted from (A) PartiGene, (B) OakContigV1 and (C) MIRA unigene elements**. Sixteen combinations of percentage of similarity (horizontal axis) and coverage (four lines) between two sequences were plotted.

BLASTClust, a part of BLAST package [20], found 114,977 peptide clusters at 70% coverage and 75% similarity for the 132,406 OakContigV1 FrameDP-predicted peptides, which corresponded to 14.2% reduction in the total number of predicted peptides (Figure 5B). Even with the 100% coverage and 100% similarity, 1,651 peptides clustered into 719 clusters. Those peptide sequences in the same cluster showed complete identity. When we performed BLASTClust analysis for 189,171 FrameDP-predicted peptides from MIRA assembly plus 'debris', there were 2,188 clusters (6,339 elements), in which all of the cluster members showed the same peptide sequence. At the 70% coverage and 75% similarity, the rate of unique peptide was 67.1% (corresponding to 32.9% reduction) (Figure 5C), which was smaller than that found in OakContigV1 (85.8%). Because reduction rate was higher using MIRA, this analysis suggests that MIRA is more efficient to distinguish not only polymorphisms and substitutions but also splice variants in the assembly step. This partly explains the difference in the depth of contigs. Contigs by MIRA had an average depth of 13.3, while that of OakContigV1 was 22.4. The BLASTClust result for PartiGene unigene elements (Figure 5A) showed similar trend to that of OakContigV1.

All together, the comparison of the procedures that were tested to assemble Sanger and 454-reads resulted into the following conclusions: first, there was an added value (in terms of integration of 454-reads) to perform a combined analysis of 454 and Sanger reads compared to a simple mapping procedure of the 454 data onto a Sanger unigene set, second, a seeded assembly using TGICL was found to be more efficient than a direct assembly using MIRA because i/ MIRA excluded a great number of ESTs from the unigene set (so called "debris"), most (67.4%) corresponding to singletons in the TGICL assembly and ii/ TGICL produced less contigs (69.2 k vs. 113.6 k) with higher depth (22.4 vs. 13.3 reads on average) and longer length (705 vs. 671 bp on average).

### Similarity searches

**Figure 6:**
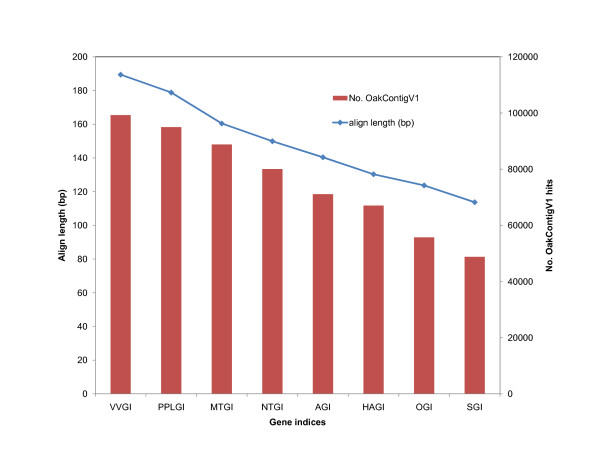
**Number of hits and high-scoring segment pair aligned length of BlastN (OakContigV1) against gene indices**. The e-value cut-off was set at 1e-3. The gene indices abbreviations are as follows: AGI; *Arabidopsis thaliana*, HAGI; *Helianthus annuus*, NTGI; *Nicotiana tabacum*, MTGI; *Medicago truncatula*, OGI; *Oryza sativa*, PPLGI; *Populus*, SGI; *Picea *and VVGI; *Vitis vinifera*.

**Figure 7:**
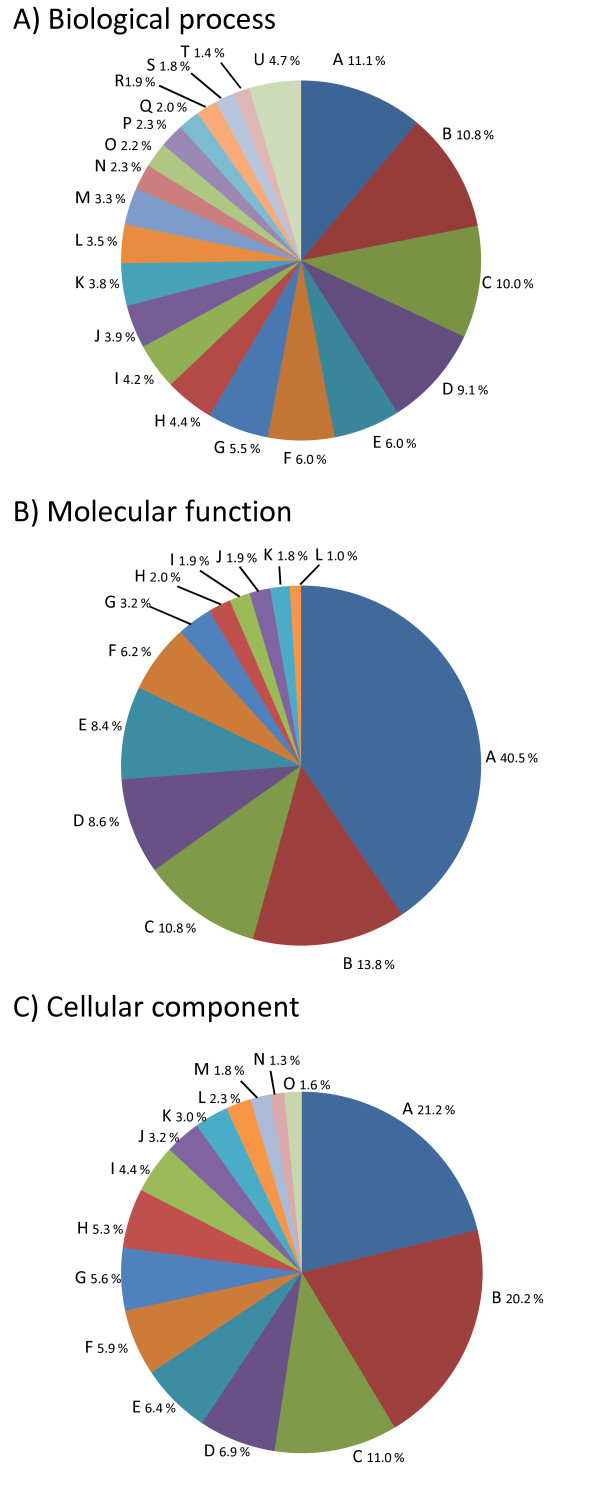
**Gene ontology classification of OakContigV1 using GO slim terms of plants**. GO terms were assigned by BlastX against SWISS_PROT database with e-value cut-off of 1e-5. GO slim terms are as follows for Biological process (A): A, Transport; B, Response to stress; C, Catabolic process; D, Protein modification process; E, Carbohydrate metabolic process; F, Transcription; G, Signal transduction; H, Cellular amino acid and derivative metabolic process; I, Translation; J, Generation of precursor metabolites and energy; K, Response to abiotic stimulus; L, Lipid metabolic process; M, Response to endogenous stimulus; N, Cell death; O, Secondary metabolic process; P, Response to biotic stimulus; Q, Cell cycle; R, Photosynthesis; S, DNA metabolic process; T, Cell differentiation; U, Others (Embryonic development, Cellular homeostasis, Cell growth, Flower development, Regulation of gene expression, epigenetic, Pollen-pistil interaction, Ripening, Response to extracellular stimulus, Tropism, Cell-cell signaling, Behavior and Abscission), for Molecular function (B) as follows: A, Nucleotide binding; B, Kinase activity; C, Transporter activity; D, Receptor activity; E, RNA binding; F, Structural molecule activity; G, Transcription factor activity; H, Nuclease activity; I, Carbohydrate binding; J, Enzyme regulator activity; K, Translation factor activity, nucleic acid binding; L, Others (Motor activity, Chromatin binding, Receptor binding, Oxygen binding and Sterol carrier activity) and for Cellular component (C) as follows: A, Plastid; B, Plasma membrane; C, Mitochondrion; D, Cytosol; E, Ribosome; F, Endoplasmic reticulum; G, Thylakoid; H, Cell wall; I, Golgi apparatus; J, Nucleolus; K, Cytoskeleton; L, Peroxisome; M, Nucleoplasm; N, Endosome; O, Others (Nuclear envelope, Lysosome, Extracellular space and Proteinaceous extracellular matrix).

Similarity searches were carried out using the hybrid assembly resulting from the TGICL pipeline (OakContigV1) that provides an approximate estimate of unique transcripts, because it discriminates alternative spliced transcripts. Out of 222,671 elements of OakContigV1, homology search against protein databases resulted into 32,810 (14.7%), 52,959 (23.8%) and 37,262 (16.7%) elements with at least one hit against SWISS-PROT [21], RefSeq_protein [22] and Pfam [23] database, respectively at the e-value cut-off of 1e-5, while that against nucleotide databases resulted in 93,658 (42.1%) and 143,830 (64.6%) unigene elements with at least one hit against Refseq_RNA and eight TIGR gene indices [24], respectively. The result of BlastN against eight gene indices showed that both the number of hits and aligned length of the high-scoring segment pair (HSP) were the greatest for sequences in VVGI (*Vitis vinifera*) and least in SGI (*Picea *sp.) (Figure 6). This may partly reflects the phylogenetic position of *Quercus *within the eurosids I. In total, 150,063 (67.4%) of OakContigV1 sequences had at least one hit in this homology search process, while the remaining sequences (72,608, i.e. 32.6%) were orphans, which may be considered as oak specific. However, caution should be made to consider orphan sequences as oak specific without experimental validation of such sequences in cDNAs. Gene ontology (GO) [25] annotation assigned at least one GO term for 29,303 (13.2%) of OakContigV1 sequences. The average number of GO annotations per sequence was 5.08, while the maximum number of annotation per sequence was 46. The total number of GO terms was 4,960. When these terms were mapped onto plant specific GO slim terms, the number of term converged to 69 terms (Figure 7). The most abundant GO slim terms were Transport, Nucleotide binding, Plastid, in terms of Biological process, Molecular function and Cellular component, respectively. Candidate genes of ecological or economic importance were found in OakContigV1 as illustrated for bud phenology (additional file 6: Table S3), drought stress resistance with emphasis on cuticle formation (additional file 7: Table S4) and phenylpropanoid biosynthesis (additional file 8: Figure S4). Genes relating to cell wall formation were detected based on tBlastX searches against MAIZEWALL database [26] (Additional file 9: Table S5). These results demonstrate the value of the EST catalogue that was produced for future functional genomics studies in oaks.

To further analyse the added value of Sanger reads in terms of annotation, we compared the annotation rate of 454 and Sanger unigene elements. From the 40,542 contigs and 139,444 singletons containing 454-reads only, 5,404 (13.3%) contigs and 33,047 (23.7%) singletons did not show a single Blast hit, whereas, from the 28,612 contigs and 14,073 singletons containing at least one Sanger read, these numbers drop down to 391 (1.37%) contigs and 1,351 (9.60%) singletons. This result clearly indicates the value of Sanger reads for functional annotation. Therefore it can be concluded that Sanger reads improve not only the assembly but also the annotation of large dataset produced by next generation technology. The fact that the lower annotation (no blast hit) rate of unigene elements containing 454-reads only may also suggest they contain higher rate of novel or artifactual transcripts. Tedersoo et al. [27] indicated that singletons from 454-reads contained higher rate of artifactual reads. Further laboratory work and/or bioinformatic characterization may be needed for the validation of singletons in OakContigV1.

### In silico mining of Simple Sequence repeats (SSRs) and Single Nucleotide Polymorphisms (SNPs)

**Figure 8:**
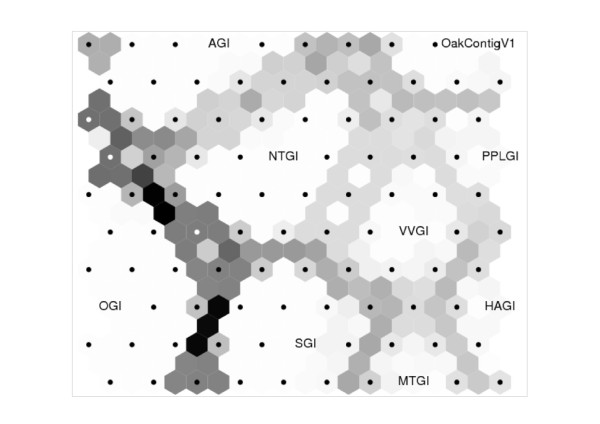
**Self organizing map for microsatellite motif distribution between eight gene indices and OakContigV1**. The gene indices abbreviations are as follows: AGI; *Arabidopsis thaliana*, HAGI; *Helianthus annuus*, NTGI; *Nicotiana tabacum*, MTGI; *Medicago truncatula*, OGI; *Oryza sativa*, PPLGI; *Populus*, SGI; *Picea *and VVGI; *Vitis vinifera*.

Using mreps [28], we found 52,834 SSRs (microsatellites) with minimum repeat of five, four, three, three and three for di-, tri-, tetra-, penta- and hexa-SSRs, respectively, in 38,653 unigene elements of OakcontigV1. Specific information for each SSR included the unigene element ID and the annotation, the repeat motif, its length and position (Additional file 10: Table S6, also available through the Quercus portal https://w3.pierroton.inra.fr:8443/QuercusPortal/Home.jsf). Dinucleotide as well as trinucleotide motifs were frequent, summing up 72.9% of the total number of microsatellites (Table 10). Among dinucleotide and trinucleotide repeats, AG and AAG motifs, respectively, were the most frequent. Only 40 CG repeats were found. Tetranucleotide (10.5%), pentanucleotide (6.8%) and Hexanucleotide (9.9%) repeats were of low abundance. The frequency of microsatellites was 23.7% considering multiple occurrence in a same unigene element, which was close to that calculated by Durand et al. [29] for 28,024 Sanger unigene elements in oak (18.6%). When we screened microsatellites within eight TIGR gene indices [24] used in the similarity search (see Method section) with the same method (*ie*. mreps), the most frequent motif was tri-SSRs (Additional file 11: Figure S5), which confirmed the general trend in SSR frequency for plant ESTs [30]. It should be noted, however, that definition of microsatellite and detection algorithm have great impact on number of detected microsatellite in silico [31]. When the distribution of SSR motif was visualized by SOM (Self Organizing Map), OakContigV1 located near PPLGI (*Populus*) (Figure 8), which may again reflect the phylogenetic position of oaks in the eurosid I. When SSR locations (coding or non-coding) were estimated by combining results from ESTScan [32] and mreps as in Durand et al. [29], the location for 38,649 (73.2%) SSRs was estimated and the same trends were found (Additional file 12: Figure S6). In brief, tri-SSRs were the most frequently found in coding regions (33.4% of the total SSRs with location estimation), while di-SSRs were frequent (27.2%) in non-coding regions. Because of functional constraints of peptides, tri-SSRs with no frame shift mutations are preferable for coding regions [33]. As discussed in [27], di-SSRs in non-coding regions were more frequently found in 5' UTRs of plant transcripts [34], suggesting that they may be involved in gene expression regulation.

**Table 10:** In silico mining of microsatellites within OakContigV1

Motif		Number of microsatellites	Percentage
	AG	13510	
	AT	3199	
	AC	2401	
	CG	40	
		
Dinucleotide	Sub-total	19150	36.25%

	AAG	5181	
	ACC	2784	
	AAC	2445	
	ATC	2195	
	AAT	2161	
	AGG	1510	
	AGC	1495	
	CCG	667	
	ACT	525	
	ACG	392	
		
Trinucleotide	Sub-total	19355	36.63%

Tetranucleotide		5520	10.45%
Pentanucleotide		3579	6.77%
Hexanucleotide		5230	9.90%

	Total	52834	100.00%

The minimum repeat number of five, four, three, three and three for di-, tri-, tetra-, penta- and hexa-microsatellites, respectively, was applied.

SNP detection was carried out on a subset of the 69,154 contigs. We first took into account the presence of duplicated reads in order to avoid false SNP detection [35,36], i.e. a single representative was kept for the analysis. Then, putative SNPs were screened for contigs with a coverage depth of more than six sequences. If the less frequent allele count was more than two and 100% identical for four bases before and after the polymorphic site, we considered this site as a putative SNP. The putative SNPs were identified among 13,334 (19.2%) contigs, resulting in 36,411 sites and an average of 1 SNP every 471 bp in contigs with putative SNPs. Average and maximum number of SNPs detected in a contig was 2.7 and 19, respectively. Transition type SNPs (A/G and T/C) were relatively frequent and amounted to 67.18% (Table 11). Within FrameDP-predicted peptides, there were 48,247 SNPs, which resulted in 27,762 (57.54%) SNPs in coding regions, including 17,620 (36.52%) and 10,140 (21.02%), synonymous and non-synonymous SNPs, respectively. SNP density in oak was lower compared to that found in *Eucalyptus grandis *transcriptomes (1 SNP every 192 bp) based on 21 individuals using GS Reference Mapper (454 Life Science, Branford, CT, USA) [37]. If we apply the same criteria to calculate SNP frequency to that of the Eucalyptus study (SNP called within contig length >200 bp and contig depth >10), the frequency remained identical (1 SNP every 471 bp), though we used more than 200 individuals for the sequencing step. In a *de novo *assembly of a coral larval transcriptome with 454 GS-FLX pyrosequencing [38], SNPs were screened by QualitySNP program and 33,433 SNPs were identified resulting in 1 SNP per 207 bp. The oak SNP frequency was still lower, probably due to more stringent criteria used for SNP detection. Using information from the predicted peptides by FrameDP (only one peptide predicted for each unigene element to avoid chimeric elements) and clustering by BLASTClust (at 70% coverage and 75% similarity), a set of 20,826 SNPs, including 16,196 and 4,630 potential coding and non-coding SNPs, respectively, was selected. We also found 59 SNPs relating to chloroplast (45) and mitochondrial (14) sequences. After these SNPs were eliminated, 20,810 genomic SNPs were retained for future genetic study. This SNP data set has also been made available for downloading at the Quercus portal https://w3.pierroton.inra.fr:8443/QuercusPortal/Home.jsf.

**Table 11:** In silico mining of SNPs within OakContigV1

SNP type	Allele	Number of SNPs	Percentage
Transition	A/G	11757	32.29%
	G/C	12703	34.89%
Transversion	A/C	2814	7.73%
	A/T	3898	10.71%
	T/G	2814	7.73%
	G/C	2313	6.35%
Tri-nucleotide		112	0.31%

Total		36411	100.00%

Synonymous (a)		17622	36.52%
Non-synonymous (b)		10140	21.02%
Coding (a)+(b)		27762	57.54%
Non-coding		20485	42.46%

Total		48247	100.00%

Peptides were predicted by FrameDP, which often produces multiple peptide for a single unigene elements. Because location of SNP sites (coding/non-coding) were estimated for each predicted peptide, the sum of coding and non-coding SNPs exceeded the total number (36,411) of SNPs. Tri-nucleotides are polymorphic sites with three alleles.

Gene diversity was calculated for 308 and 1,770 SNP sites within *Q. robur *and *Q. petraea*, respectively (for criteria to select SNP sites for gene diversity calculation, see Methods section). The averages were 0.3336 and 0.3340 for *Q. robur *and *Q. petraea*, respectively. These values were comparable to those calculated from marker-based analysis for *Cryptomeria japonica *(*H_e _*= 0.322) [39] and *Eucalyptus grandis *and *E. smithii *(*PIC *= 0.357) [40].

### Detection of orthologous and paralogous gene pairs between oak and the eudicotyledons sequenced reference genomes

Recently, Salse et al. [41] published an original and robust method for the identification of orthologous regions between plant genomes as well as for the detection of duplications within genomes based on integrative sequence alignment criteria combined with a statistical validation. This approach was applied to identify 7 paleo-duplications in monocots and eudicots and to propose a common ancestor with 5 and 7 chromosomes for the monocots and eudicots respectively [42]. In the current study, we used the 31,798 unigene set resulting from the PartiGene assembly and FrameDP analysis to integrate the oak transcriptome information into previous paleo-genomics analysis in order to unravel the oak evolutionary paleo-history.

**Figure 9:**
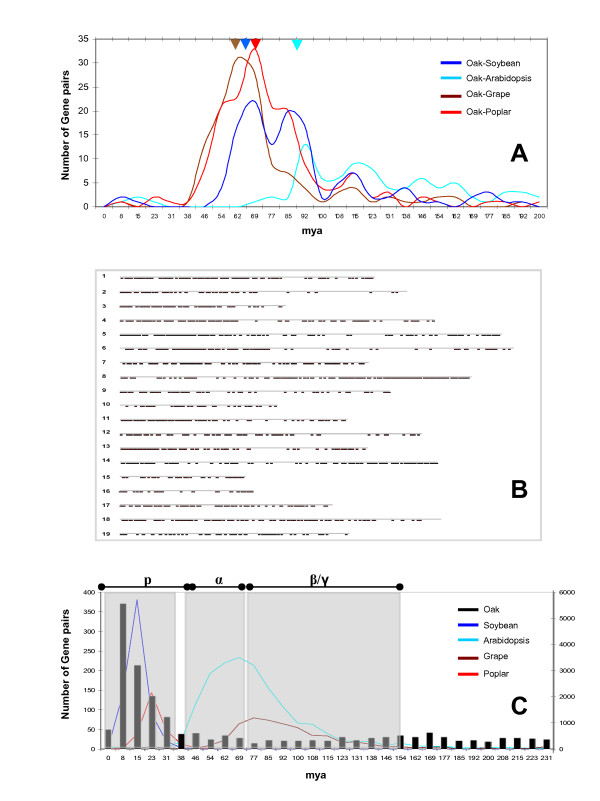
**Oak genome orthologous and paralogous relationships**. A. The distribution of Ks distance (scaled in MYA) values observed for the orthologous gene pairs identified between oak and *Arabidopsis *(light blue curve), poplar (red curve), grape (brown curve) and soybean (blue curve) genomes are illustrated as number of syntenic gene pairs (y-axis) per dating intervals (x-axis). Distribution peaks are highlighted with colored arrows. B. Schematic representation of the heterologous oak gene map illustrating the 1,825 orthologs identified between oak and grape and positioned on the 19 grape chromosomes. C. The distribution of Ks distance (scaled in MYA) values observed for the paralogous gene pairs identified for the oak (black bars), *Arabidopsis *(light blue curve), poplar (red curve), grape (brown curve), soybean (blue curve) genomes are illustrated as number of duplicated gene pairs (y-axis, left scale for oak/*Arabidopsis*/grape and right scale for poplar/soybean) per dating intervals (x-axis). The distinct rounds of whole genome duplication (p, α, β, γ) reported for the eudicot genome paleo-history are highlighted with grey boxes.

Using the alignment parameters and statistic tests previously described by Salse et al. [42,43], we analysed the orthologous relationships between oak, Arabidopsis (33,198 gene models), poplar (30,260 gene models), grape (21,189 gene models) and soybean (46,194 gene models) genomes. Based on the 31,798 oak unigene elements, we identified 4,574 orthologous gene pairs between oak and *Arabidopsis *(477 orthologs), poplar (658 orthologs), grape (1,825 orthologs) and soybean (1,614 orthologs) genomes. The Ks distribution analysis (Figure 9A) performed between the 4,574 orthologous gene pairs establishes that oak is most closely related at the sequence level to grape (brown curve and arrow) than any other eudicot genome included in the analysis. We then produced a heterologous oak gene map based on the precise identification of oak orthologs on the 19 grape chromosomes (Figure 9B). This 1,825 robust orthologs identified between oak and grape can be considered as a valuable source of COS (Comparative Orthologous Sequences) markers for further comparative genomics and genetics analysis [43].

We applied the most robust and direct approach allowing the characterization of genome duplications that consists of aligning the available unigene set (31,798 elements) on itself using stringent alignment criteria and statistical validation described in Salse et al. [43]. The Ks distribution analysis (Figure 9C) obtained for 1,526 (43%) of the 3,520 paralogous gene pairs available (black bars) when compared to that obtained for *Arabidopsis *(1,646 paralogs, light blue curve), poplar (4,164 paralogs, red curve), grape (542 paralogs, brown curve), and soybean (9,532 paralogs, blue curve) genomes clearly established that the actual oak genome went through at least two rounds or series of whole genome duplications (grey boxes on Figure 9C), such as ancestral (referenced as γ in the literature based on grape distribution peak in brown) shared by the eudicots and more recent (referenced as α/β based on the *Arabidopsis *distribution peak in light blue or p based on soybean and poplar distribution peaks respectively in dark blue and red).

## Conclusions

We collected about 146 K Sanger and 2 M 454-ESTs for two oak species: *Q. petraea *and *Q. robur*. Seeded assembly by TGICL software produced 222,671 unigene elements (OakContigV1: 69,154 contigs and 153,517 singletons). On the one hand, the 454-pyrosequencing data contributed to greatly increased the number of unigene elements compared to that obtained by assembling Sanger reads only (40,944 unigene elements). On the other hand, Sanger reads significantly improved the quality of the assembly both in terms of clustering and annotation

Based on similarity searches, we identified candidate genes for traits of ecological importance as well as comparative orthologous sequences (COS) which were mapped onto *Vitis *(grape) chromosomes. These COSs may be considered as a valuable source of genetic markers for comparative genomic analysis. Evolutionary analysis also showed that grape was the closest to oak within the eudicots. Additional data mining within OakContigV1 identified 52,834 SSRs and 36,411 putative SNPs that can be used as functional markers in future studies. These resources are publically available from the oak contig browser at http://genotoul-contigbrowser.toulouse.inra.fr:9092/Quercus_robur/index.html (user: oak, pass word: quercus33).

This large collection of expressed sequence tags provide an important resource for the scientific community that is interested in the molecular genetics and functional genomics of Oaks. It is a fundamental resource for analysis of gene expression, discovery of genes of ecological interest, comparative mapping, and annotation of the forthcoming oak genome sequence.

## Methods

### Plant material for Sanger sequencing

Plant material used to generate the cDNA and SSH libraries were collected from different tissues, developmental stages or after different treatments. Below we summarize (into six categories) the 20 libraries that were produced for *Q. robur *and *Q. petraea *(see Table 1):

i/ libraries H and I: *Quercus petraea *seedlings were grown at INRA Nancy (North East of France). Acorns were harvested in a local forest and sown in 10 L containers with a peat and sand mixture (1/1: v/v). A complete fertilisation (4.5 g L^-1 ^of slow-release fertiliser Nutricote T100; N/P/K/Mg: 13/13/13/2 + trace elements and 0.2 g L^-1 ^of lime) was provided at the beginning. All individuals were watered daily to field capacity with deionized water. The seedlings were 22-24 weeks old when submitted to five different treatments. Three different seedlings were submitted to each stress. For the cold (10°C for 3 days) and heat (35°C for 4 days) stress treatments, growth cabinets were used. The elevated CO_2 _treatment (700 ppm for 18 days) was done using a climate controlled greenhouse. For the drought stress treatment, irrigation was stopped, the soil humidity was measured daily using a TDR (Trase 6050X1, Soilmoisture Equipment Corp., Santa Barbara CA, USA) and seedlings were harvested at about 17% remaining soil humidity. For root hypoxia, the seedlings were placed into water tight containers and the water level was maintained 1 cm above soil level. All stresses were pooled and three biological replicates were extracted and mixed in equal amount for cDNA synthesis.

ii/ libraries B, J, K, L, P, S: adult trees from South West of France (INRA Pierroton forestry station) were sampled in their natural area. Leaves were taken in summer, differentiating xylem in spring and vegetative buds both in winter and spring to provide different bud development stages from the same genotypes.

iii/ libraries A, C, D, E: vegetative buds from one- or two-year old seedlings from North West of France were acclimated in the nursery of INRA Pierroton (South West of France).

iv/ library M: roots from one-year old trees were grown under optimal conditions in a greenhouse at INRA Nancy.

v/ libraries Q, R: second flush sun-leaves were harvested in greenhouse grown plants in summer 2004 on one-year old cuttings from high and low water-use efficient genotypes identified in Brendel et al. [44].

vi/ libraries F, G, N, O, T: cuttings were obtained from 10 sessile and 10 pedunculate oak trees in August 2006 at INRA Pierroton. After three months of growth, cuttings were placed into oxygenated water tight containers for 2 weeks in a growth chamber providing a 16-h photoperiod, a day/night temperature of 25°C/20°C, a day/night relative humidity of 85%/70% and a quantum flux of 260 μmol m^-2 ^s^-1^. Root hypoxia was then imposed by using deoxygenated water obtained by bulling with N_2_, in order to maintain the O_2 _concentration below 5 mg L^-1^. White roots from each genotype and species were sampled after 6, 24 and 48 hours of hypoxia. In all cases, white roots were immediately dipped in liquid nitrogen to prevent degradation and stored at -80°C until RNA extraction.

### RNA extraction for Sanger sequencing

Oak material was collected from either field grown trees or seedlings raised in greenhouse or phytotrons. Plant material used as RNA source for cDNA and subtractive library construction was detailed above. For each library devoted to Sanger sequencing, total RNA was extracted following the procedure described by Le Provost et al. [45] with a final purification step using the RNAeasy kit (QIAGEN, Courtaboeuf, France).

### cDNA library construction for Sanger sequencing

Depending on the quantity of each tissue, cDNA libraries were constructed using either CloneMiner cDNA Library Construction Kit (Invitrogen Corporation, Carlsbad, CA, USA) and Stratagene cDNA synthesis kit (Stratagene, La Jolla, CA, USA) when plant material was abundant, or Creator SMART cDNA Library Construction Kit (Clontech Laboratories Inc., Mountain View, CA, USA) when plant material was limiting.

For libraries constructed with CloneMiner kit, mRNA was isolated from 200 μg of total RNA of each genotype using Dynabeads mRNA Purification Kit (Invitrogen Corporation) according to the manufacturer's protocol. cDNA synthesis was performed using 2 μg of mRNA. Ligation to attB1 adapter, cDNA size-fractionation and recombination reaction between pDONR222 vector and cDNAs were performed as described in the User Manual. Finally, ElectroMax DH10B T1 Phage Resistant Cells (Invitrogen Corporation) were transformed with recombinant plasmids by electroporation. Each library titer was estimated on Kanamycin LB plates (50 μg mL^-1^). Libraries were stored at -80°C in glycerol Super Optimal broth with Catabolite repression (SOC) medium until sequencing.

Libraries constructed using the Stratagene kit were done according to the manufacturer's recommendations. The resulting cDNAs were packaged into λ ZAP II phages using the Gigapack III Gold packaging kit (Stratagene).

For libraries constructed with Creator kit, equal quantities of total RNA from each genotype were mixed and cDNA synthesis was then performed from 1 μg of total RNA by long distance PCR according to the manufacturer's instructions. Sfi1 digestion, cDNA size fractionation, plasmid ligation and transformation into ElectroMax DH10B T1 Phage resistant cells were done according to the manufacturer's protocol. An aliquot of each transformation was spread on Chloramphenicol LB plates (30 μg mL^-1^) to determine the percentage of recombinant clones. When more than 75% of recombinant clones were obtained, transformation mixtures were pooled together to constitute the library. If necessary, new ligations were performed to give a final library of approximately 10^6 ^clones: the titer was estimated on Chloramphenicol LB plates. Glycerol SOC medium was added and libraries were stored at -80°C.

For suppression subtractive hybridization libraries, we used the method, originally described by Diatchenko et al. [46], that is based on selective amplification of differentially expressed sequences. All libraries were comprised of a unique tissue collected on several genotypes of *Quercus *species. Total RNA was extracted using the method described above. Double-stranded tester cDNA and driver cDNA were prepared from 1 μg total RNA of each sample using the SMART™PCR cDNA Synthesis Kit (Clontech) according to the manufacturer's protocol. The forward subtracted libraries were constructed using the PCR-select cDNA Subtraction Kit (Clontech). Amplified, differentially expressed cDNA fragments were cloned into either the pGEM T easy vector (Promega, Madison, WI, USA) or the PCR4 TOPO kit from Invitrogen. ESTs were obtained from the following tissues:

• Bud: three SSH libraries (C, D, E), obtained as detailed in Derory et al. [47]

• Leaf: two SSH libraries (Q, R) obtained using total RNA extracted from leaves sampled on 5 genotypes displaying extreme phenotypes for water-use-efficiency (WUE). The libraries were obtained by subtracting RNA form High WUE phenotypes vs. Low WUE phenotypes, and *vice-versa *[48].

• Root: four SSH libraries (N and O for *Q. robur*, F and G for *Q. petraea*) constructed by subtracting sessile against pedunculate mRNA and *vice-versa *for early (6 hours) and late (24 and 48 hours pooled) flooding stress.

### Sanger DNA sequencing

Sequencing was completed using the standard Sanger method as described by Sanger et al. [49]. Briefly, clones were randomly isolated and arranged individually in 384-well microtitre plates for storage and processing and subjected to high-throughput single-path sequencing from either their 5'- and/or 3'-ends. cDNA libraries were sequenced at "Centre National de Séquençage" (Genoscope, Evry, France). SSH libraries were sequenced at the "Genome & Transcriptome" facility of Bordeaux. Briefly recombinant clones were re-amplified using M13 universal primer. PCR products were then purified using Multiscreen PCR micro 96 kit (Millipore, San Francisco, CA, USA) and subjected to single pass sequencing from their 5'-and/or 3'-end using BigDye version 3.1 kit (Applied Biosystems, Foster city, CA, USA) according to the manufacturer's instructions. All the sequences were run on an ABI 3730 (Applied Biosystems, Foster city, CA, USA) sequencing machine.

### Plant material for pyrosequencing

Plant material was collected on different genotypes (Table 4):

i/ libraries I to IV: buds were taken from two populations (30 *Q. petraea *genotypes each) at endo- and eco-dormancy to provide clues about genes differentially expressed between these two developmental stages.

ii/ libraries V and VI: leaves and buds were sampled from two species (*Q. robur *and *Q. petraea*, 10 genotypes each) for the discovery of genes involved in species divergence.

iii/ libraries VII and VIII: pollen and flowers were collected on 2 *Q. petraea *and 2 *Q. robur *genotypes, to enrich the tissue panel with reproductive organs.

iv/ libraries IX to XIV: buds and leaves were collected on 6 parental trees of three full-sub pedigrees to detect polymorphic markers for genetic linkage mapping.

### RNA extraction for pyrosequencing

Total RNA was extracted as described by Le Provost et al. [45]

### cDNA library construction for pyrosequencing

We used the SMART PCR cDNA synthesis kit (Clontech) and MINT cDNA synthesis Kit (Evrogen) according to manufacturer's instructions. Library normalization was done for libraries IX to XIV by Beckman Coulter Genomics (Grenoble, France) using Duplex-Specific-Nuclease (Evrogen) according to manufacturer's instructions.

### 454-sequencing

cDNA nebulisation, adaptor ligation, emulsion PCR and sequencing were done at Beckman Coulter Genomics (Danvers, MA, USA). Sequencing was performed using a Roche-454 Genome Sequencer platform (FLX or Titanium technology).

### Sequence processing

We have summarized the approach that was followed in Figure 1 (blue boxes).

A total of 145,827 Sanger ESTs were cleaned using the **S**eq**U**ence **R**epository and **F**eature detection pipeline (SURF, http://surf.toulouse.inra.fr/cgi-bin/surf.cgi, user name: oak, password: oak1). The documentation of SURF can be found at http://genome.jouy.inra.fr/doc/bioinfo/edition/surf-1.0/SURF.pdf. SURF includes: i/ base calling by phred [12,13], ii/ masking and clipping of library specific vectors/adaptors using cross_match [14] with -minmatch 10 -minscore 15, iii/ masking low complexity regions (mononucleotide repeats) using RepeatMasker [50], iv/ screening of PCR kit sequences using cross_match against short UniVec ftp://ftp.ncbi.nih.gov/pub/UniVec/ sequences with parameters -minmatch 8 -minscore 10, v/ screening and elimination of possible contaminants in putative insert using cross_match on the UniVec with -minmatch 10 -minscore 25 and short UniVec with -minmatch 10 -minscore 15, as well as on *E. coli *and yeast sequences with -minmatch 100 -minscore 150, and vi/ detection of chloroplast sequences using cross_match with -minmatch 100 -minscore 150. The *Quercus robur *chloroplast genome sequence was kindly provided by F Sebastiani and GG Vendramin from CNR (Florence, Italy). The detailed parameters used in SURF are documented on the SURF web site. Three other features, namely "doubtful", "pcrkitful" and "not valid" were added by SURF. If library specific vectors, adaptors and primers were detected inside an insert, SURF judged the sequence as "doubtful" (possible chimera), if SURF detected short UniVec sequences inside an insert, the sequence was labelled as "pcrkitful" and if SURF detected contaminants inside an insert, "not valid" status was attached to the sequence. Too short sequences (length < 100) with high quality (phred QV > 20) were discarded for further analysis.

Prior to submission of sequences to the EMBL database, reads were further processed by qualityTrimmer in Euler-SR package [51] with the option -minQual 20. As indicated in Table 2, a total of 125,886 ESTs were finally submitted to EMBL: 57,750 for *Q. petraea *and 68,136 for *Q. robur*. These sequences can be accessed by submitting the following query: Quercus [Organism name] and Frigerio [Authors] to EMBL. Quality scores can be downloaded from SURF web site.

Sanger reads were also processed by trace2dbEST (ver. 3.0.1) pipeline [11] using the default parameters except that the minimum sequence length, required for sequences to pass further analysis, was set at 60 bp. The phred [12,13] parameter for error probability was set to 0.05 (default value). The cross_match [14] parameters for vector (including adaptor) and *E. coli *masking were set at -minmatch 10 -minscore 20 and -minmatch 20 and -minscore 30, respectively. These cross_match parameters corresponded to the default values for trace2dbEST. Screening libraries, which included any sequences of adaptors, adaptor and vector junctions and vectors, were constructed for each vector and adaptor combinations. Poly A/T sequences with repeats ≥12 were also screened and masked. Possible chimera sequences were suggested by trace2dbEST if vector and/or *E. coli *sequences were detected in the middle of an insert. They were then discarded from further analysis.

The 454-reads (1,948,579 sequences) were screened by cross_match [14] for primers and adaptors and then masked. For each 454-read, the longest non-masked region was extracted and further cleaned-up by SeqClean [52]. The shorter regions were discarded in order to take care of potential chimeras. This process resulted into 1,578,192 clean reads. The sequencing statistics for 454-reads were calculated by NG6 system (http://vm-bioinfo.toulouse.inra.fr/ng6/, user name: oak, password: quercus33). SRA (Sequence Read Archive) accession number is SRA012448 and can be accessed at http://www.ncbi.nlm.nih.gov/sra. In NG6, four kinds of analysis were performed in parallel for each library: i/ in the first analysis, contaminants were searched for *E. coli*, phage and yeasts with Blast, ii/ in the second, the quality of each read was analyzed, iii/ in the third, reads were analyzed by pyrocleaner, to remove too short or too long sequences (sequences with more than or less than two standard deviations from the mean), dirty sequences (sequences with more than 4% of N), low complexity sequences and duplicated reads [35,36], and iv/ in the fourth, reads were assembled by Newbler (Roche) within each library. The duplicated reads in step iii were defined as clusters by megablast with minimum hit score of 100, percent identity cut-off of 98, alignment of reads starting exactly at the same position and ending in a 70 bp window of the end of the longest sequence.

### Coverage of transcripts within libraries

In order to estimate the coverage of transcripts within libraries, we first randomized links between a EST sequence and the corresponding contig. Secondly, for each library, 100 EST sequences were selected and the number of corresponding contigs was counted. The second step was repeated until no EST sequences were left for the library. The relationship between number of EST sequences (X) and the number of contigs (Y) were modelled by the following equation:

Y=A{1−exp(BX)}

where A and B are coefficients determined by nls function of R language http://www.r-project.org/. The coefficient A indicates the expected maximum number of contigs (transcripts) in a library under this model. The coefficient B as well as A relates to the rate of the increase of the number of contigs. The library coverage was defined as the ratio of the observed number of contigs by the coefficient A. We excluded libraries C, D, E, G, N, Q and R for this analysis, because they did not contain enough ESTs.

### Sequence assembly

Assembly of Sanger and 454-reads was first carried out using the SIGENAE system http://www.sigenae.org/ that is based on the TGICL software http://compbio.dfci.harvard.edu/tgi/software/[16]. This software uses the CAP3 assembler [53] that takes into account the quality of sequenced nucleotides into the computation of the alignment score. The different steps of the assembly are highlighted in red in Figure 1. First, Sanger reads were assembled into 15,835 tentative consensus sequences (TC) using TGICL (mgblast, a modified version of megablast [54] and CAP3). The software FrameDP [19] was then used to predict complete ORF of 2,000 TC (the longest ones) with SWISS-PROT [21] as a reference, resulting in 224 TCs which potentially contain full-length coding sequences (starting with a methionine residue and ending with a stop codon). The TCs were used to split large clusters built by mgblast using the sclust program within TGICL. Global assembly was performed using TGICL with 125,925 Sanger ESTs, 224 TCs and the 1,578,192 454-ESTs with sequence qualities and options of (-l) minimum overlap length of 100, (-p) minimum percent identity for overlap of 96 and (-s) splitting clusters larger than 100,000. The resulting 222,671 contigs and singletons were called OakContigV1 and are available at http://genotoul-contigbrowser.toulouse.inra.fr:9092/Quercus_robur/index.html (user: oak, password: quercus33). Because all of the sequences resulted from assembly were directly loaded into the OakContigV1 database, low quality regions were not filtered out.

MIRA (V3rc4) software [18,55] was also used to directly perform hybrid assembly of Sanger and 454-reads (Figure 1, purple boxes), instead of assembling the consensus of 454 data with Sanger reads as in TGICL. MIRA was run with a standard options (-job = denovo, est, normal, sanger, 454) and no XML files. The assembly by MIRA was compared with OakContigV1. Contigs showing Reciprocal best Blast Hit (RBH) was searched between sequences in OakContigV1 and MIRA assembly by using BlastN with "soft" filtering option. Soft filtering makes it efficient to detect orthologous sequences [56].

We also used the PartiGene (ver. 3.0.5) pipeline [15] to compare OakContigV1 assembly to the assembly constructed using Sanger reads only (Figure 1, green boxes). The sequences cleaned by trace2dbEST were grouped, using CLOBB [57], into clusters based on Blast similarity. Sequences in the same cluster were then assembled using Phrap [14] (using the default parameters of PartiGene). The resulting 40,944 unigene elements (23,730 singletons and 17,214 contigs) were used for peptide prediction using FrameDP [19]. The resulting 36,883 peptide sequences were finally used to detect comparative orthologous markers (see section below). Assemblies within each Sanger library were also conducted by PartiGene to estimate redundancy rate of libraries. The assembly by PartiGene was compared with OakContigV1. Contigs showing RBH was searched between sequences in OakContigV1 and PartiGene assembly by using BlastN with "soft" filtering option [56].

Finally, MIRA (V3.0.0) software was used to map 454-reads to the unigene elements constructed by PartiGene with the following options (job = mapping, normal, 454) and no XML files for 454-reads.

### Annotation and similarity searches

A functional annotation was assigned for each contig and singleton of Oakcontigv1 (Figure 1, gray boxes). The strategy is based on homology search with public protein and nucleic acid sequence databases. BlastX [20] was carried out against SWISS-PROT (Release 57.1 of 14-Apr-2009) [21] and RefSeq_protein (Release 34 of 6-March-2009) [22] with e-value cut-off at 1e-5. Conserved protein domains were searched against Pfam (Release 23.0 of 20-Jul-2008) [23] with e-value cut-off at 1e-5. Blastn was carried out against OakContigV1, Refseq_RNA (Release 34 of 6-March-2009) and TIGR gene indices with e-value cut-off of 1e-30, 1e-5 and 1e-2, respectively. We used the following TIGR gene indices [24]: AGI (Arabidopsis_thaliana release_14), HAGI (Helianthus_annuus release_6), NTGI (Nicotiana_tabacum release_5), MTGI (Medicago_truncatula release_9), OGI (Oryza_sativa release_17), PPLGI (Populus release_4), SGI (Picea release_3) and VVGI (Vitis_vinifera release_6). These gene indices were selected so as to represent phylogenetic relationships of land plants as follows: gymnosperm (SGI), monocots (OGI), rosids (VVGI), rosid I (MTGI and PPLGI), rosid II (AGI), asterid I (NTGI) and asterid II (HAGI). The Gene Ontology (GO) [25] annotation was based on the best hit in SWISS-PROT. The GO terms were mapped upon plant GOslim terms using Blast2GO software [58].

### Detection of unique peptides based on FrameDP peptide prediction

Because oak ESTs contain sequences from about 200 individuals of *Q. robur *and *Q. petraea*, we expected to detect not only polymorphisms within species but also substitutions between species in the combined assembly. In addition, ESTs were collected from multi-stressed libraries (Table 1), which are likely to increase the number of splice variants. These factors may split EST clusters into multiple contigs. In order to estimate the minimum unigene sets, FrameDP [19] was used, translating assembled sequences into peptide sequences (Figure 1, orange boxes). The TAIR9_pep sequences (available at ftp://ftp.arabidopsis.org/home/tair/Sequences/blast_datasets/) were used as reference sequences in FrameDP. The resulting peptide sequences from OakContigV1 were further clustered by BLASTClust, a part of the BLAST package [20] to provide a set of unique peptide that was further used to retain *in silico *SNPs within coding sequences.

### Identification of candidate genes

Candidate genes for bud phenology related genes (list kindly provided by M. Lascoux & G. Zaina) were searched against peptide sequences estimated by FrameDP. The predicted peptide sequences were used in BlastP with e-value cut-off set at 1e-5. Drought stress resistance candidate genes with emphasis on cuticle formation in *Arabidopsis thaliana *were searched within OakContigV1 by BlastP or BlastX with e-value cut-off of 1e-10. Genes of the phenylpropanoid pathway within OakContigv1 were also targeted. We used GO annotation (see previous section) to convert GO terms into enzyme code (EC) using Blast2GO software [58]. Those EC corresponding to the phenylpropanoid pathway were mapped on the corresponding KEGG map [59]. In order to detect genes related to cell wall formation in OakContigV1, tBlastX was performed against the MAIZEWALL database [26] with e-value cut-off of 1e-10.

### *In silico mining *of SSRs and SNPs

To detect simple sequence repeats (SSRs), we used mreps [28] and listed microsatellites with minimum repeat number of five, four, three, three and three for di-, tri-, tetra-, penta- and hexa-SSRs, respectively. SSRs were also screened within eight gene indices used for the annotation (see the section "Annotation and similarity searches"). To visualize phylogenetic similarity of the SSR motif distribution, data were analyzed by self organizing map (SOM) using som_pack (ver. 3.1) [60], which utilizes unsupervised pattern recognition algorithms.

For SNP detection, we first took into account the presence of duplicated reads in order to avoid false SNP detection [35,36], i.e. a single representative was kept for the analysis. Then, putative SNPs were screened for contigs with a coverage depth of more than six sequences. If the less frequent allele count was more than two and 100% identical for four bases before and after the polymorphic site, we considered this site as a putative SNP. SNPs were summarized according to the following categories: transition/transversion, synonymous/non-synonymous and coding/non-coding. The frame-corrected nucleotide sequences inferred by FrameDP [19] as coding regions were used for the identification of "coding SNPs". By fasty35 program in FASTA package [61], we re-mapped the frame-corrected nucleotide sequence onto corresponding OakContigV1 original sequence. The peptide sequences inferred by FrameDP were used as references to identify non-synonymous mutations at the SNP site. Using tfasty35, non-synonymous mutations were identified by alignment of the allelic peptide sequence with the reference peptide sequence. Finally, a more stringent set of SNPs was retained, considering contigs for which a single protein was predicted by FrameDP. In addition, SNPs whose unigene elements represented significant blast hits with organelle sequences (*Q. robur *chloroplast kindly provided by F Sebastiani and GG Vendramin from CNR, Florence, Italy and *Vitis vinifera *mitochondria [62]) were detected. The percent identity and coverage threshold was set at 90% and 80%, respectively, for chloroplast and 60% and 70%, respectively, for mitochondria. These SNPs useful for population genetic studies were discarded from the nuclear SNP data set. Both nuclear and organelle SNPs data sets have been made available at Quercus portal https://w3.pierroton.inra.fr:8443/QuercusPortal/Home.jsf. SNPs whose unigene elements presented Sanger reads with significant hit with *Q. robur *chloroplast sequence in the SURF process were also eliminated.

Gene diversity ($H_{E}=n(1-\sum_{i}p_{i}^{2})/(n-1)$), where n is the number of reads included for the calculation and *P_i _*is allele frequency, was estimated for *Quercus*. At SNP sites in each assembly, we randomly selected one read from each library to avoid multiple sampling of the same allele in the same individual. We only targeted SNP sites with number of reads ≥8 for each species.

### Evolutionary analysis

#### Arabidopsis, grape, poplar and soybean sequence databases

The Arabidopsis (5 chromosomes - 33,198 genes - 119 Mb - ftp://ftp.arabidopsis.org/home/tair/Genes/TAIR9_genome_release/TAIR9_sequences/), Grape (19 chromosomes - 21 189 genes - 302 Mb - http://www.genoscope.cns.fr/externe/Download/Projets/Projet_ML/data/), Poplar (19 chromosomes - 30 260 genes - 294 Mb - ftp://ftp.jgi-psf.org/pub/JGI_data/Poplar/), Soybean (20 chromosomes - 46 194 genes - 949 Mb - ftp://ftp.jgi-psf.org/pub/JGI_data/phytozome/v5.0/Gmax/) genome sequences were used for comparative genomics study.

### Synteny and duplication analysis

We used the two parameters recently defined by Salse et al. [41-43] to increase the stringency and significance of Blast sequence alignment by parsing Blast results and rebuilding HSPs (High Scoring Pairs) or pairwise sequence alignments to identify accurate paralogous and orthologous relationships.

### Distribution of K_S _distances (MYA scale) for paralogous and ortholougous gene pairs

We performed the sequence divergence as well as speciation event datation analysis based on the rate of nonsynonymous (*Ka*) vs. synonymous (*Ks*) substitutions calculated with PAML (Phylogenetic Analysis by Maximum Likelihood) [63]. The average substitution rate (r) of 6.5 × 10^-9 ^substitutions per synonymous site per year for grasses is classically used to calibrate the ages of the considered gene [64,65]. The time (*T*) since gene insertion is then classically estimated using the formula *T *= *Ks*/r.

## Authors' contributions

SU analysed the data and wrote the manuscript. CP conceived the study, coordinated the ForEST project and wrote the manuscript. GLP, VL, CP, PA and OB prepared the biological material. GLP, JD, VL, FS and PA constructed the libraries. CK and CN supervised the bioinformatic analysis (SURF, NG6, TGICL) and implemented the data into a powered Ensembl database. JS, MA and FM performed the evolutionary analysis. JMF, CC, AB and ADD managed the ESTs produced within the EVOLTREE and ForEST projects and submitted the ESTs to public databases. PW, AC, PL took care of the SANGER sequencing. AK coordinated the Evoltree project and was assisted by MPR. All authors read and approved the final version of the manuscript.

## Supplementary Material

Additional file 1 **Table S1: Number of reads with significant Blast hits against *E. coli*, phage and yeast sequences for libraries pyrosequenced by Roche 454**. Click here for file

Additional file 2 **Table S2: Newbler assembly in NG6 **http://vm-bioinfo.toulouse.inra.fr/ng6/**for libraries pyrosequenced by Roche 454**. Click here for file

Additional file 3 **Figure S1: Sequence length distribution for unigene elements constructed by (A) PartiGene, (B) TGICL and (C) MIRA**. Unigene elements (contigs and singletons) by PartiGene (A) were from Sanger reads only, while those by MIRA (B) and TGICL (C) were from both Sanger and 454-reads. The unigene elements by TGICL is named as "OakContigV1". Click here for file

Additional file 4 **Figure S2: Distribution of the number of reads in a contig (depth of a contig)**. Contigs resulting from PartiGene (brown bar), TGICL (green bar) and MIRA (blue bar) analysis. Click here for file

Additional file 5 **Figure S3: Frequency distribution of the number of peptides predicted from unigene elements**. Frequency of FrameDP-predicted peptides resulting from PartiGene (brown bar), TGICL (green bar) and MIRA (blue bar) assembly. Click here for file

Additional file 6 **Table S3: Oak homologs to poplar candidate genes for bud phenology**. Click here for file

Additional file 7 **Table S4: Oak homologs with *Arabidopsis thaliana *for drought stress resistance related genes with emphasis on cuticle formation**. Click here for file

Additional file 8 **Figure S4: Phenylpropanoid biosynthesis related genes found in OakContigV1**. List of genes are as follows with the number of OakContigV1 sequences in parenthesis. Red; EC:2.1.1.104 [caffeoyl-CoA O-methyltransferase] (31), Yellow; EC:1.11.1.7 [peroxidase] (212), Orange; EC:1.1.1.195 [cinnamyl-alcohol dehydrogenase] (28), Green; EC:3.2.1.21 [beta-glucosidase] (54), Blue; EC:2.1.1.68 [caffeate O-methyltransferase] (38), Pink; EC:2.3.1.92 [sinapoylglucose---malate O-sinapoyltransferase] (1), Violet; EC:2.3.1.91 [sinapoylglucose---choline O-sinapoyltransferase] (2), Light-red; EC:1.2.1.68 [coniferyl-aldehyde dehydrogenase] (3), Light-green; EC:1.14.13.11 [trans-cinnamate 4-monooxygenase] (10), Light-yellow; EC:6.2.1.12 [4-coumarate---CoA ligase] (15). Click here for file

Additional file 9 **Table S5: Homology search results against MAIZEWALL database**. Click here for file

Additional file 10 **Table S6: SSRs detected in OakContigV1 sequences**. SSR motifs (5, 4, 3, 3, and 3 repeats at least for di-, tri-, tetra-, penta- and hexa-nucleotides, respectively) were searched by mreps (Kolpakov et al. 2003) program to detect microsatellite repeats from OakContigV1. Annotations are based on BlastX search against SWISS-PROT database with e-value cut-off 1e-5. "nil" indicates no hits. Click here for file

Additional file 11 **Figure S5: Microsatellite frequency detected by mreps for eight gene indices and OakContigV1**. The search was performed for di-(with a repeat count n >= 5 repeat units), tri- (n >= 4), tetra- (n >= 3), penta- (n >= 3) and hexa- (n >= 3) nucleotides. The gene indices abbreviations are as follows: AGI; *Arabidopsis thaliana*, HAGI; *Helianthus annuus*, NTGI; *Nicotiana tabacum*, MTGI; *Medicago truncatula*, OGI; *Oryza sativa*, PPLGI; *Populus*, SGI; *Picea *and VVGI; *Vitis vinifera*. Click here for file

Additional file 12 **Figure S6: Estimation of SSR location by analysis with ESTScan and mreps software**. Click here for file
