# Supplementary material for: Bioinformatic analysis of ESTs collected by Sanger and pyrosequencing methods for a keystone forest tree species: oak
Source: BMC Genomics. 2010 Nov 23;11:650. doi: 10.1186/1471-2164-11-650 (PMC3017864; doi:10.1186/1471-2164-11-650)
Supplement: Additional file 1 — Table S1: Number of reads with significant Blast hits against E. coli, phage and yeast sequences for libraries pyrosequenced by Roche 454. [file 1471-2164-11-650-S1.PDF]

Table S1. **Number of reads with significant Blast hits against *E. coli*, phage and yeast sequences for libraries pyrosequenced by Roche 454**

Library codes are as in Table 4.

Library code	454	*E. coli*	Phage	Yeast
I	FLX	0	0	0
II	FLX	0	0	1
III	FLX	0	0	0
IV	FLX	0	0	2
V	FLX	0	0	3
VI	FLX	0	0	0
VII	FLX	0	0	3
VIII	FLX	0	0	4
IX	Titanium	73	45	1
X	Titanium	83	25	1
XI	Titanium	134	71	0
XII	Titanium	93	63	4
XIII	Titanium	99	35	3
XIV	Titanium	96	45	0
