# Supplementary material for: Bioinformatic analysis of ESTs collected by Sanger and pyrosequencing methods for a keystone forest tree species: oak
Source: BMC Genomics. 2010 Nov 23;11:650. doi: 10.1186/1471-2164-11-650 (PMC3017864; doi:10.1186/1471-2164-11-650)
Supplement: Additional file 2 — Table S2: Newbler assembly in NG6 http://vm-bioinfo.toulouse.inra.fr/ng6/for libraries pyrosequenced by Roche 454. [file 1471-2164-11-650-S2.PDF]

Table S2. **Newbler assembly in NG6 (http://vm-bioinfo.toulouse.inra.fr/ng6/) for libraries pyrosequenced by Roche 454**

Library codes are as in Table 4.

Library code	454	Number of reads (a)	Number of large contig	Number of total contigs (b)	Number of singletons (c)	Average contig length (bp)	Average depth	Unique sequence rate ((b)+(c))/(a)
I	FLX	115050	541	4126	53201	345.83	4.95	49.8%
II	FLX	137380	883	5833	58399	356.33	5.6	46.8%
III	FLX	79345	361	2869	32299	339.82	4.71	44.3%
IV	FLX	164140	1486	8373	54758	379.03	5.52	38.5%
V	FLX	159478	1843	7667	60554	439.98	5.01	42.8%
VI	FLX	99472	965	4664	46195	403.5	4.93	51.1%
VII	FLX	112207	838	5839	50512	357.24	4.92	50.2%
VIII	FLX	154819	1385	7578	60642	389.9	5.07	44.1%
IX	Titanium	153558	5775	8280	24739	774.93	7.97	21.5%
X	Titanium	124143	5179	7267	22402	772.53	7.32	23.9%
XI	Titanium	206828	7357	10290	24606	785.78	10.08	16.9%
XII	Titanium	137409	4891	6702	19711	816.1	8.58	19.2%
XIII	Titanium	143969	5003	6797	18898	816.33	9.05	17.8%
XIV	Titanium	160781	5295	7559	24919	798.44	7.81	20.2%
