# Supplementary figures and images for: Bioinformatic analysis of ESTs collected by Sanger and pyrosequencing methods for a keystone forest tree species: oak
Source: BMC Genomics. 2010 Nov 23;11:650. doi: 10.1186/1471-2164-11-650 (PMC3017864; doi:10.1186/1471-2164-11-650)

## Slide 1
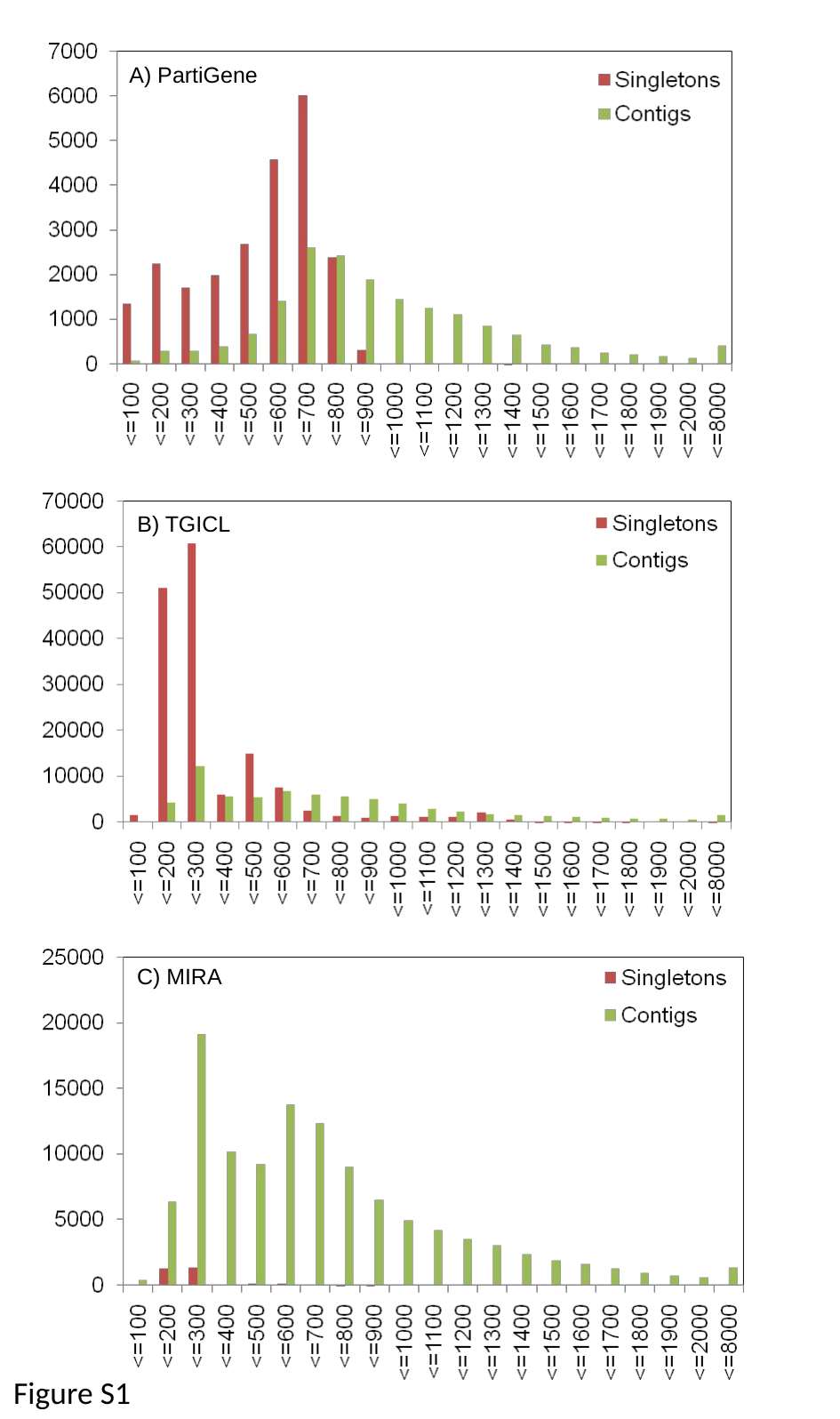

A) PartiGene
B) TGICL
C) MIRA
Figure S1

Supplement: Additional file 3 — Figure S1: Sequence length distribution for unigene elements constructed by (A) PartiGene, (B) TGICL and (C) MIRA. Unigene elements (contigs and singletons) by PartiGene (A) were from Sanger reads only, while those by MIRA (B) and TGICL (C) were from both Sanger and 454-reads. The unigene elements by TGICL is named as "OakContigV1". [file 1471-2164-11-650-S3.PPT]

## Slide 1
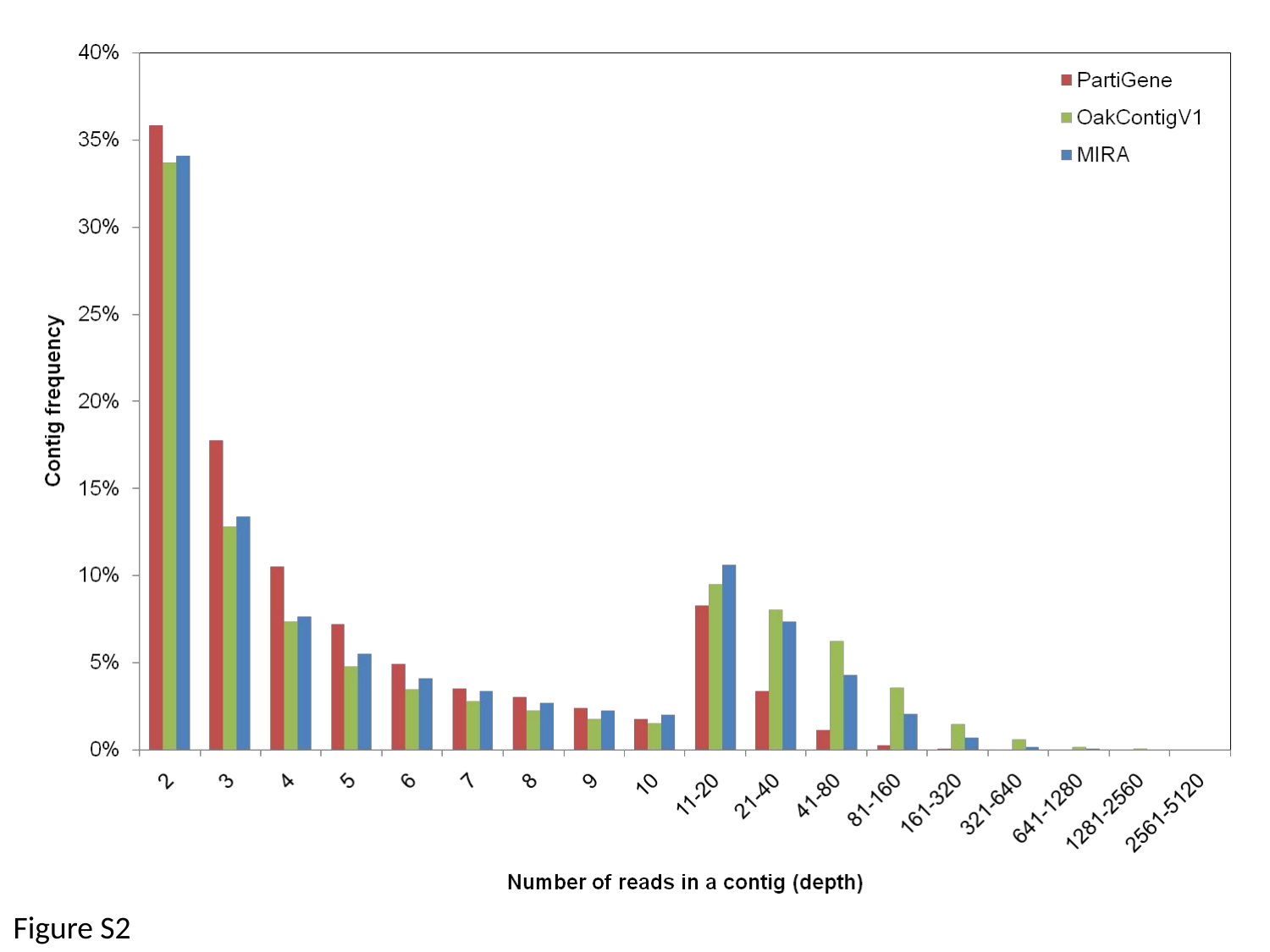

Figure S2

Supplement: Additional file 4 — Figure S2: Distribution of the number of reads in a contig (depth of a contig). Contigs resulting from PartiGene (brown bar), TGICL (green bar) and MIRA (blue bar) analysis. [file 1471-2164-11-650-S4.PPT]

## Slide 1
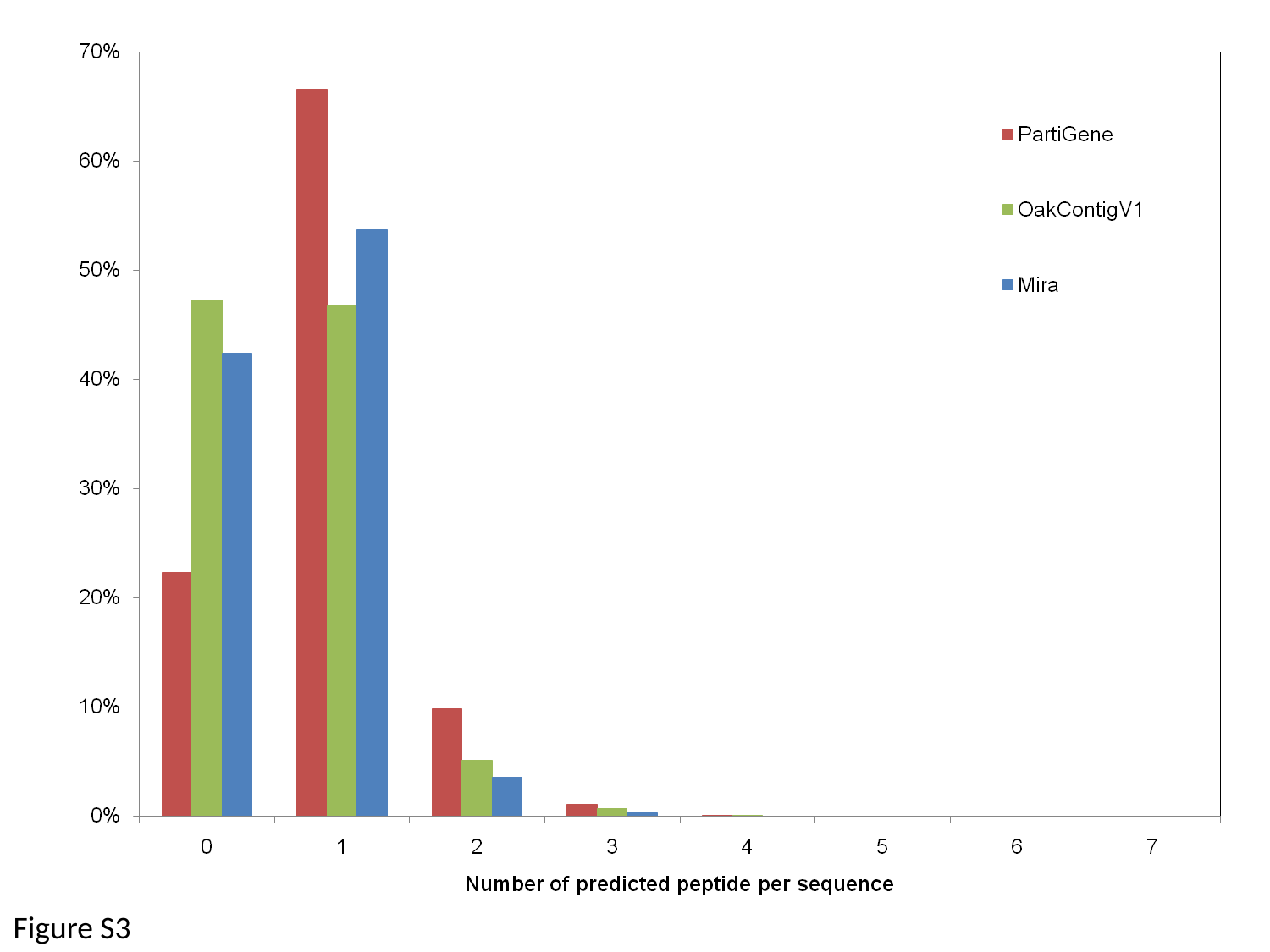

Figure S3

Supplement: Additional file 5 — Figure S3: Frequency distribution of the number of peptides predicted from unigene elements. Frequency of FrameDP-predicted peptides resulting from PartiGene (brown bar), TGICL (green bar) and MIRA (blue bar) assembly. [file 1471-2164-11-650-S5.PPT]

## Slide 1
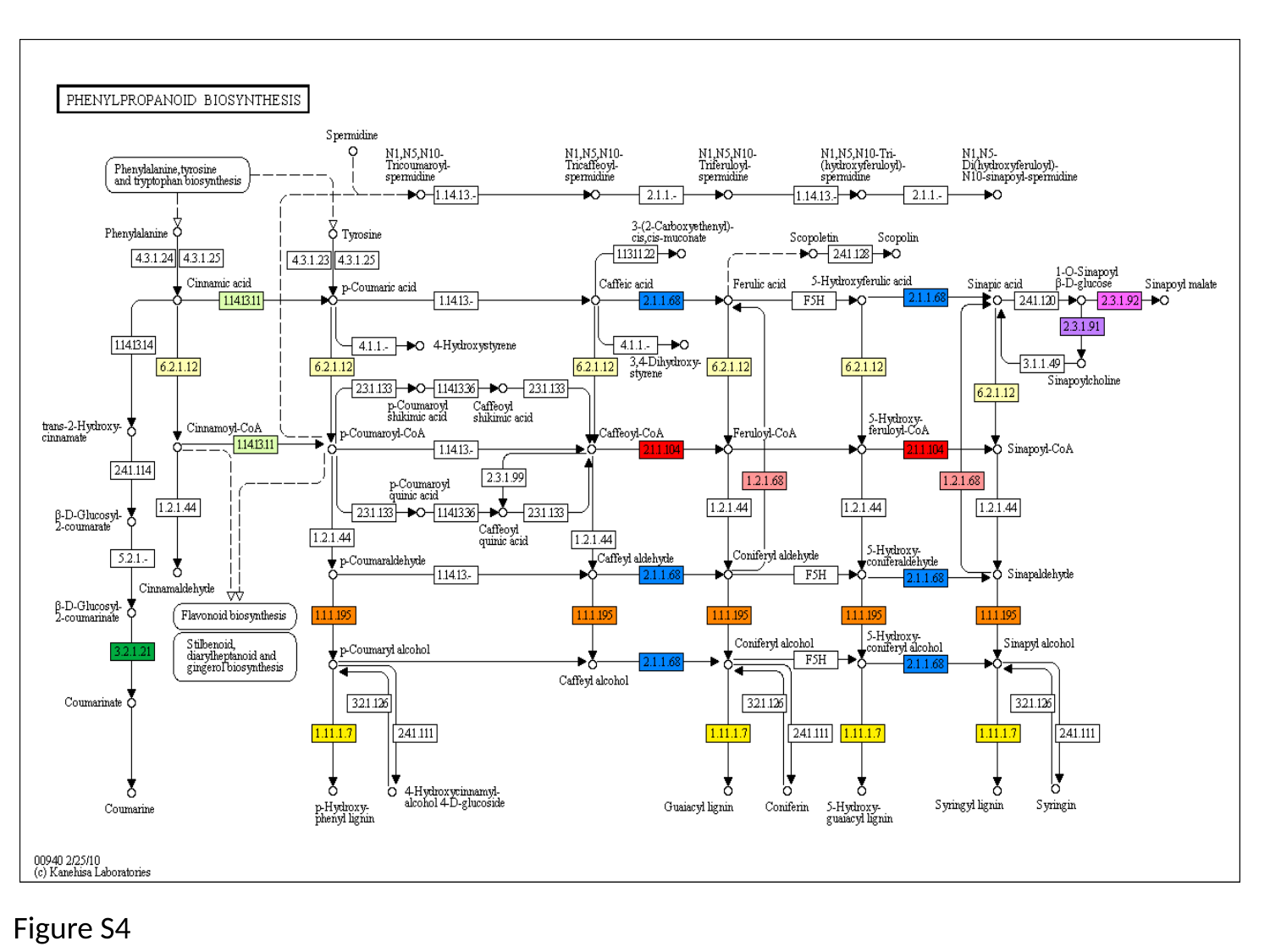

Figure S4

Supplement: Additional file 8 — Figure S4: Phenylpropanoid biosynthesis related genes found in OakContigV1. List of genes are as follows with the number of OakContigV1 sequences in parenthesis. Red; EC:2.1.1.104 [caffeoyl-CoA O-methyltransferase] (31), Yellow; EC:1.11.1.7 [peroxidase] (212), Orange; EC:1.1.1.195 [cinnamyl-alcohol dehydrogenase] (28), Green; EC:3.2.1.21 [beta-glucosidase] (54), Blue; EC:2.1.1.68 [caffeate O-methyltransferase] (38), Pink; EC:2.3.1.92 [sinapoylglucose---malate O-sinapoyltransferase] (1), Violet; EC:2.3.1.91 [sinapoylglucose---choline O-sinapoyltransferase] (2), Light-red; EC:1.2.1.68 [coniferyl-aldehyde dehydrogenase] (3), Light-green; EC:1.14.13.11 [trans-cinnamate 4-monooxygenase] (10), Light-yellow; EC:6.2.1.12 [4-coumarate---CoA ligase] (15). [file 1471-2164-11-650-S8.PPT]

## Slide 1
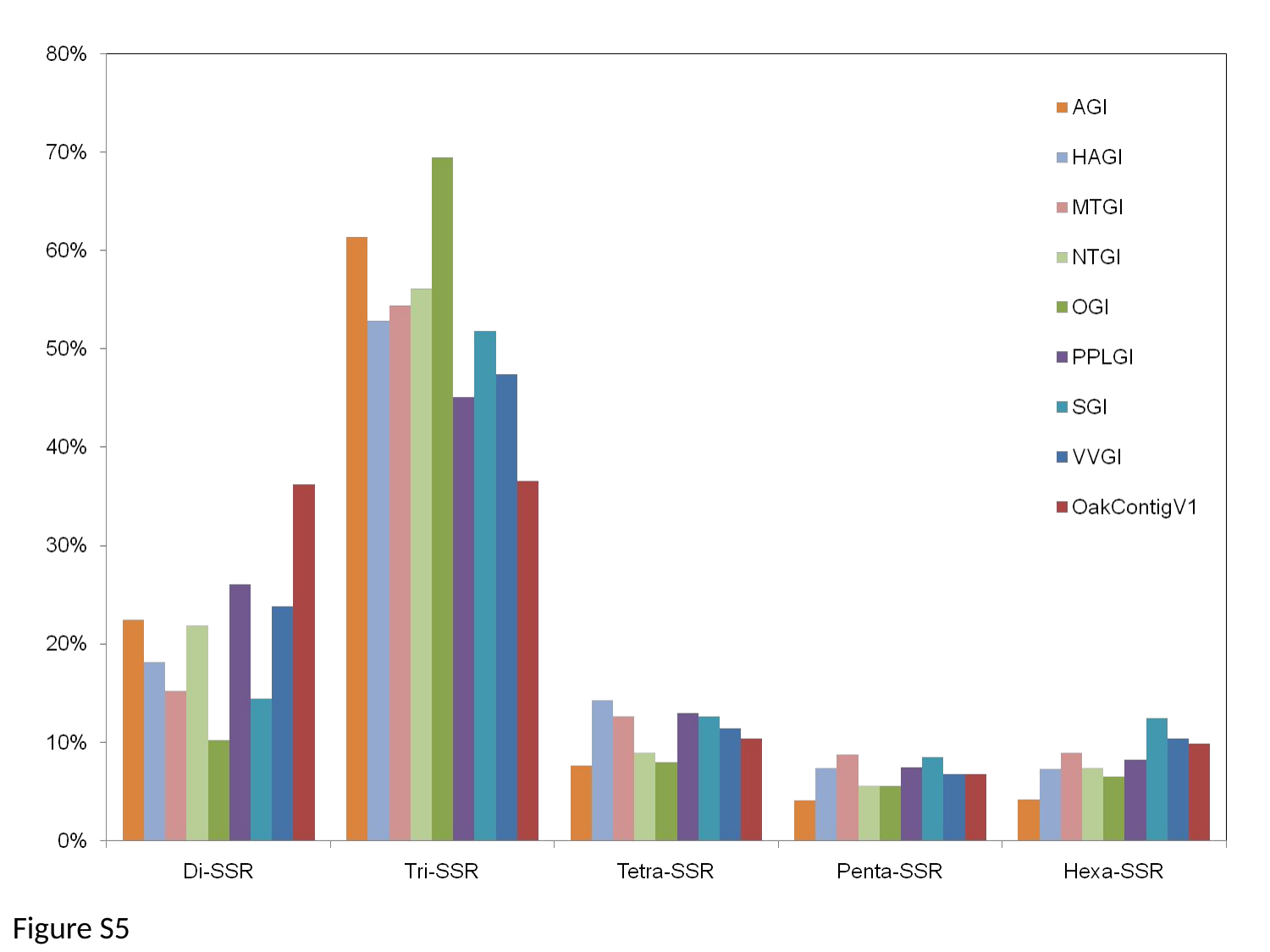

Figure S5

Supplement: Additional file 11 — Figure S5: Microsatellite frequency detected by mreps for eight gene indices and OakContigV1. The search was performed for di-(with a repeat count n >= 5 repeat units), tri- (n >= 4), tetra- (n >= 3), penta- (n >= 3) and hexa- (n >= 3) nucleotides. The gene indices abbreviations are as follows: AGI; Arabidopsis thaliana, HAGI; Helianthus annuus, NTGI; Nicotiana tabacum, MTGI; Medicago truncatula, OGI; Oryza sativa, PPLGI; Populus, SGI; Picea and VVGI; Vitis vinifera. [file 1471-2164-11-650-S11.PPT]

## Slide 1
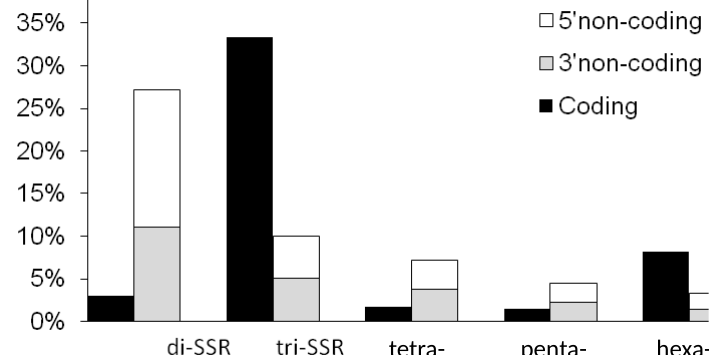

tetra-SSR
penta-SSR
hexa-SSR
Figure S6

Supplement: Additional file 12 — Figure S6: Estimation of SSR location by analysis with ESTScan and mreps software. [file 1471-2164-11-650-S12.PPT]
