# Supplementary material for: Bioinformatic analysis of ESTs collected by Sanger and pyrosequencing methods for a keystone forest tree species: oak
Source: BMC Genomics. 2010 Nov 23;11:650. doi: 10.1186/1471-2164-11-650 (PMC3017864; doi:10.1186/1471-2164-11-650)
Supplement: Additional file 6 — Table S3: Oak homologs to poplar candidate genes for bud phenology. [file 1471-2164-11-650-S6.PDF]

Table S3. Oak homologs to poplar candidate genes for bud phenology

Gene	Query name (JGI or DDBJ Accession)	Top hit oak peptide ID	HSP % identity	HSP evalue
Phytochrome A	jgi|Poptr1_1|729311|estExt_Genewise1_v1.C_LG_XIII0395	F0SUT5C01A3J3U.l.qr.1:1:1865:3	83.5	0
Phytochrome B1	jgi|Poptr1_1|832686|estExt_fgenesh4_pm.C_LG_VIII0434	F0SUT5C01A3J3U.l.qr.1:1:1865:3	51.1	1E-180
Phytochrome B2	jgi|Poptr1_1|1091155|estExt_Genewise1Plus.C_LG_X3762	F0SUT5C01A3J3U.l.qr.1:1:1865:3	50.9	1E-163
Cryptochrome 1	jgi|Poptr1_1|830225|estExt_fgenesh4_pm.C_LG_II0442	F0SUT5C01BHSIH.l.qr.1:202:2247:1	87.0	0
Cryptochrome 2	jgi|Poptr1_1|281234|gw1.273.26.1	F0SUT5C01ASI01.l.qr.1:138:1526:3	83.9	0
Cryptochrome 3	jgi|Poptr1_1|803751|fgenesh4_pm.C_LG_VIII000706	F0SUT5C01ASI01.l.qr.1:138:1526:3	70.0	0
Cryptochrome 4	jgi|Poptr1_1|559103|eugene3.00050718	F0SUT5C01BHSIH.l.qr.1:202:2247:1	85.9	0
Constans-like 1	jgi|Poptr1_1|266027|gw1.123.49.1	F0SUT5C01BPYQH.l.qr.1:1:1258:2	75.2	1E-159
Constans-like 2	jgi|Poptr1_1|831202|estExt_fgenesh4_pm.C_LG_IV0339	F0SUT5C01BPYQH.l.qr.1:1:1258:2	75.5	1E-164
Suppressor of Constans 1	jgi|Poptr1_1|244198|gw1.XIV.941.1	F0SUT5C02G8P4G.l.qr.1:130:783:1	76.4	1E-82
Frigida	jgi|Poptr1_1|253112|gw1.XV.2548.1	F0SUT5C01AJ2TD.l.qr.1:1:1567:2	76.5	2E-32
PhyA signal transduction factor 1a	jgi|Poptr1_1|246964|gw1.XIV.3707.1	F0SUT5C01A240P.l.qr.1:1:1175:3	78.8	1E-177
PhyA signal transduction factor 1b	jgi|Poptr1_1|730290|estExt_Genewise1_v1.C_LG_XIII2678	F0SUT5C01BS6HU.l.qr.1:293:1570:2	69.8	0
PhyA signal transduction factor-like LGI	jgi|Poptr1_1|176020|gw1.I.4620.1	F0SUT5C01A240P.l.qr.1:1:1175:3	79.6	1E-180
PhyA signal transduction factor-like LGXVI	jgi|Poptr1_1|575909|eugene3.00160044	F0SUT5C01BTAI0.l.qr.1:356:2032:2	65.5	0
PhyA signal transduction factor-like LGVI	jgi|Poptr1_1|652427|grail3.0024010601	F0SUT5C01BTAI0.l.qr.1:356:2032:2	64.3	0
Gigantea 1	jgi|Poptr1_1|818606|estExt_fgenesh4_pg.C_LG_V1131	F0SUT5C01A3EE8.l.qr.1:73:2220:1	81.6	0
Gigantea 2	jgi|Poptr1_1|551288|eugene3.00020603	F0SUT5C01A3EE8.l.qr.1:73:2220:1	79.9	0
Timing of CAB expression 1 protein	jgi|Poptr1_1|784463|fgenesh4_pg.C_scaffold_129000038	F0SUT5C01A8BAA.l.qr.1:375:2024:3	73.3	0
TOC1-like	jgi|Poptr1_1|824063|estExt_fgenesh4_pg.C_LG_XIV0468	F0SUT5C01AVGXT.l.qr.1:199:2253:1	54.6	0
TOC1-like	jgi|Poptr1_1|422771|gw1.XII.1231.1	F0SUT5C01AVGXT.l.qr.1:199:2253:1	40.5	1E-104
TOC1-like	jgi|Poptr1_1|574629|eugene3.00150024	F0SUT5C01AVGXT.l.qr.1:199:2253:1	42.2	1E-123
Late elongated hypocotyl/CCA1	jgi|Poptr1_1|731468|estExt_Genewise1_v1.C_LG_XIV1950	F0SUT5C01AQQPW.l.qr.1:234:2402:3	64.5	0
Circadian clock coupling factor	jgi|Poptr1_1|778575|fgenesh4_pg.C_LG_XVII000454	F0SUT5C02G7C9Q.l.qr.1:198:947:3	89.9	1E-137
Circadian clock coupling factor	jgi|Poptr1_1|818049|estExt_fgenesh4_pg.C_LG_IV1139	F0SUT5C02G7C9Q.l.qr.1:198:947:3	92.7	1E-141
Transcriptional regulation complex	jgi|Poptr1_1|559406|eugene3.00051021	F0SUT5C01A4P71.l.qr.1:286:1566:1	84.5	0
Zinc finger domain-containing protein	jgi|Poptr1_1|209269|gw1.V.4670.1	F0SUT5C01A8G05.l.qr.1:218:1120:2	60.1	5E-83
Ethylene responsive element binding factor	jgi|Poptr1_1|660903|grail3.0014007501	F0SUT5C01A9GCH.l.qr.1:538:1176:1	58.9	2E-57
Protein phosphatase 2C-like	jgi|Poptr1_1|568871|eugene3.00110885	F0SUT5C01AH5XD.l.qr.1:394:1315:2	82.0	1E-133
Squamosa Promoter-binding protein	jgi|Poptr1_1|888732|e_gw1.XI.3794.1	F0SUT5C01EF2PD.l.qr.1:1:596:3	60.7	1E-50
NAC3/NAC4 protein	jgi|Poptr1_1|728432|estExt_Genewise1_v1.C_LG_XI3994	F0SUT5C01A04VD.l.qr.1:114:1343:3	71.1	1E-135
Flowering time locus 1a member	jgi|Poptr1_1|575797|eugene3.00151192	F0SUT5C01AHZOO.l.qr.1:16:543:1	54.5	1E-49
Vernalization locus 2	jgi|Poptr1_1|412944|gw1.III.47.1	F0SUT5C01BP5SK.l.qr.1:1:1498:2	64.6	1E-175
Vernalization locus 2	jgi|Poptr1_1|814985|estExt_fgenesh4_pg.C_LG_I0694	F0SUT5C01BP5SK.l.qr.1:1:1498:2	66.0	0
Flowering locus T, FT1-like	jgi|Poptr1_1|765657|fgenesh4_pg.C_LG_VIII000671	F0SUT5C01BPBNU.l.qr.1:1:319:1	88.3	4E-47
Flowering locus T, FT2-like	jgi|Poptr1_1|582519|eugene3.14090001	F0SUT5C01BPBNU.l.qr.1:1:319:1	90.4	1E-49
Flowering Locus T/Terminal Flower 1	AB106111	F0SUT5C01BPBNU.l.qr.1:1:319:1	90.4	1E-49
