# Supplementary material for: Bioinformatic analysis of ESTs collected by Sanger and pyrosequencing methods for a keystone forest tree species: oak
Source: BMC Genomics. 2010 Nov 23;11:650. doi: 10.1186/1471-2164-11-650 (PMC3017864; doi:10.1186/1471-2164-11-650)
Supplement: Additional file 7 — Table S4: Oak homologs with Arabidopsis thaliana for drought stress resistance related genes with emphasis on cuticle formation. [file 1471-2164-11-650-S7.PDF]

Table S4. **Oak homologs with *Arabidopsis thaliana* for drought stress resistance related genes with emphasis on cuticle formation**

Pathway	Family	AT	function	TOPhit OakContigV1 peptide	HSP percent identity	HSP evalue
Waxes Biosynthesis	Alcohol-forming Fatty Acyl-CoA Reductase (FAR)	At3g11980	MS2;FAR2	F0SUT5C01A63J7.l.qr.1:1:1553:3	40.9	1E-101
Waxes Biosynthesis	Alcohol-forming Fatty Acyl-CoA Reductase (FAR)	At3g44540	FAR4	F0SUT5C01A63J7.l.qr.1:1:1553:3	51.5	1E-144
Waxes Biosynthesis	Alcohol-forming Fatty Acyl-CoA Reductase (FAR)	At3g44550	FAR5	F0SUT5C01A63J7.l.qr.1:1:1553:3	49.6	1E-137
Waxes Biosynthesis	Alcohol-forming Fatty Acyl-CoA Reductase (FAR)	At3g44560	FAR8	F0SUT5C01A63J7.l.qr.1:1:1553:3	48.2	1E-134
Waxes Biosynthesis	Alcohol-forming Fatty Acyl-CoA Reductase (FAR)	At3g56700	FAR7	F0SUT5C01A63J7.l.qr.1:1:1553:3	36.9	1E-78
Waxes Biosynthesis	Alcohol-forming Fatty Acyl-CoA Reductase (FAR)	At4g33790	MS2like;FAR3; cer4	F0SUT5C01A63J7.l.qr.1:1:1553:3	64.0	0
Waxes Biosynthesis	Alcohol-forming Fatty Acyl-CoA Reductase (FAR)	At5g22420	FAR6	F0SUT5C01A63J7.l.qr.1:1:1553:3	45.8	5E-95
Waxes Biosynthesis	Alcohol-forming Fatty Acyl-CoA Reductase (FAR)	At5g22500	MS2like;FAR1	F0SUT5C01A63J7.l.qr.1:1:1553:3	51.5	1E-138
Waxes Biosynthesis	Wax synthase	At1g34490		F0SUT5C01AU7KY.l.qr.1:272:1357:2	41.0	4E-67
Waxes Biosynthesis	Wax synthase	At1g34500		F0SUT5C01AU7KY.l.qr.1:272:1357:2	41.6	1E-71
Waxes Biosynthesis	Wax synthase	At1g34520		F0SUT5C01AU7KY.l.qr.1:272:1357:2	36.4	5E-43
Waxes Biosynthesis	Wax synthase	At3g51970	WS1	F0SUT5C02HO4LV.l.qr.1:1:1265:3	40.3	4E-75
Waxes Biosynthesis	Wax synthase	At5g51420		F0SUT5C02HO4LV.l.qr.1:1:1265:3	38.2	6E-62
Waxes Biosynthesis	Wax synthase	At5g55320		F0SUT5C01AU7KY.l.qr.1:272:1357:2	35.1	1E-55
Waxes Biosynthesis	Wax synthase	At5g55330	WS4	F0SUT5C01AU7KY.l.qr.1:272:1357:2	43.9	1E-79
Waxes Biosynthesis	Wax synthase	At5g55340	WS2	F0SUT5C01AU7KY.l.qr.1:272:1357:2	41.3	9E-68
Waxes Biosynthesis	Wax synthase	At5g55350		F0SUT5C01AU7KY.l.qr.1:272:1357:2	40.0	4E-72
Waxes Biosynthesis	Wax synthase	At5g55360		F0SUT5C01AU7KY.l.qr.1:272:1357:2	43.6	3E-76
Waxes Biosynthesis	Wax synthase	At5g55370		F0SUT5C01AU7KY.l.qr.1:272:1357:2	38.0	5E-66
Waxes Biosynthesis	Wax synthase	At5g55380	WS3	F0SUT5C01AU7KY.l.qr.1:272:1357:2	44.0	3E-80
Waxes Biosynthesis	CER1	At1g02190	CER1like1	F0SUT5C01A6SDH.l.qr.1:328:2214:1	33.3	1E-99
Waxes Biosynthesis	Aldehyde Decarbonylase	At1g02205	CER1	F0SUT5C01A6SDH.l.qr.1:328:2214:1	36.7	3E-87
Waxes Biosynthesis	Aldehyde Decarbonylase	At2g37700	CER1like3	F0SUT5C01A6SDH.l.qr.1:328:2214:1	31.4	4E-88
Waxes Biosynthesis	Aldehyde Decarbonylase	At5g28280	CER1like4	F0SUT5C02IKY1U.l.qr.1:1:421:2	45.9	5E-30
Waxes Biosynthesis	Aldehyde Decarbonylase CER3	At5g57800	WAX2; CER1like2	F0SUT5C01A6SDH.l.qr.1:328:2214:1	66.3	0
Waxes Biosynthesis	Aldehyde Decarbonylase CER2	At3g23840	CER2 like1	F0SUT5C01BOWWW.l.qr.1:62:1351:2	35.2	7E-68
Waxes Biosynthesis	Putative Transcription Factor	At4g24510	CER2	F0SUT5C01BOWWW.l.qr.1:62:1351:2	40.7	2E-84
Waxes Biosynthesis	Putative Transcription Factor	At4g13840	CER2 like2	F0SUT5C02ITDYU.l.qr.1:1:1088:3	38.1	2E-62
Waxes Biosynthesis	SHINE	At1g15360	SHN1	F0SUT5C01C9FTB.l.qr.1:1:803:3	52.5	5E-54
Waxes Biosynthesis	SHINE	At5g11190	SHN2	F0SUT5C01CAF0H.l.qr.1:61:678:1	57.9	1E-55

Waxes Biosynthesis	SHINE	At5g25390	SHN3	F0SUT5C01CAF0H.l.qr.1:61:678:1	62.8	2E-60
Waxes Biosynthesis	Bifunctional wax ester synthase/diacylglycérol acyltransferase	At1g72110		F0SUT5C02F3XTY.l.qr.1:1:1203:1	39.5	2E-78
Waxes Biosynthesis	Bifunctional wax ester synthase/diacylglycérol acyltransferase	At2g38995		F0SUT5C02F3XTY.l.qr.1:1:1203:1	44.4	5E-95
Waxes Biosynthesis	Bifunctional wax ester synthase/diacylglycérol acyltransferase	At3g49190		F0SUT5C02F3XTY.l.qr.1:1:1203:1	49.5	1E-107
Waxes Biosynthesis	Bifunctional wax ester synthase/diacylglycérol acyltransferase	At3g49200		F0SUT5C02F3XTY.l.qr.1:1:1203:1	50.3	1E-110
Waxes Biosynthesis	Bifunctional wax ester synthase/diacylglycérol acyltransferase	At3g49210	senescence	F0SUT5C02F3XTY.l.qr.1:1:1203:1	50.5	1E-110
Waxes Biosynthesis	Bifunctional wax ester synthase/diacylglycérol acyltransferase	At5g12420		F0SUT5C02F3XTY.l.qr.1:1:1203:1	45.4	1E-102
Waxes Biosynthesis	Bifunctional wax ester synthase/diacylglycérol acyltransferase	At5g16350		F0SUT5C02F3XTY.l.qr.1:1:1203:1	40.8	1E-88
Waxes Biosynthesis	Bifunctional wax ester synthase/diacylglycérol acyltransferase	At5g22490		F0SUT5C02F3XTY.l.qr.1:1:1203:1	44.2	3E-91
Waxes Biosynthesis	Bifunctional wax ester synthase/diacylglycérol acyltransferase	At5g37300		F0SUT5C02F3XTY.l.qr.1:1:1203:1	44.1	5E-91
Waxes Biosynthesis	Bifunctional wax ester synthase/diacylglycérol acyltransferase	At5g53380		F0SUT5C02F3XTY.l.qr.1:1:1203:1	45.9	7E-97
Waxes Biosynthesis	Bifunctional wax ester synthase/diacylglycérol acyltransferase	At5g53390		F0SUT5C02F3XTY.l.qr.1:1:1203:1	49.1	1E-111
Waxes Biosynthesis	CER7	At3g60500	Regulation	F0SUT5C02FIWYP.l.qr.1:1:1054:3	57.8	1E-106
Waxes Biosynthesis	RST1	At3g27670	Regulation	F0SUT5C02GT8BS.l.qr.1:40:726:1	58.4	4E-56
Cutin Biosynthesis	Thioesterase	At3g25210	FatA	F0SUT5C02FT1L1.l.qr.1:1:2057:3	26.7	8E-21
Cutin Biosynthesis	Thioesterase	At4g13050	FatA	F0SUT5C01A6OTM.l.qr.1:1:1220:3	67.6	1E-141
Cutin Biosynthesis	Thioesterase	At1g08510	FatB	F0SUT5C01B4R1M.l.qr.1:112:1392:1	72.6	1E-169
Cutin Biosynthesis	Acyl-CoA Synthetase	At2g47240	LACS1	F0SUT5C01A1XMS.l.qr.1:158:1681:2	69.7	0
Cutin Biosynthesis	Acyl-CoA Synthetase	At1g49430	LACS2	F0SUT5C01A8RDL.l.qr.1:72:2060:3	53.8	0
Cutin Biosynthesis	Acyl-CoA Synthetase	At1g64400	LACS3	F0SUT5C01A8RDL.l.qr.1:72:2060:3	70.9	0
Cutin Biosynthesis	Acyl-CoA Synthetase	At4g23850	LACS4	F0SUT5C01A8RDL.l.qr.1:72:2060:3	75.9	0
Cutin Biosynthesis	Acyl-CoA Synthetase	At4g11030	LACS5	F0SUT5C01A8RDL.l.qr.1:72:2060:3	73.4	0
Cutin Biosynthesis	Acyl-CoA Synthetase	At2g04350	LACS8	F0SUT5C01A32Q6.l.qr.1:1:2054:3	75.8	0
Cutin Biosynthesis	Acyl-CoA Synthetase	At1g77590	LACS9	F0SUT5C01A32Q6.l.qr.1:1:2054:3	66.4	0
Cutin Biosynthesis	Acyl-CoA Synthetase	At4g14070	AAE15	F0SUT5C01CGNAI.l.qr.1:1:523:2	69.2	1E-66
Cutin Biosynthesis	Acyl-CoA Synthetase	At3g23790	AEE16	F0SUT5C01CGNAI.l.qr.1:1:523:2	72.8	1E-71
Cutin Biosynthesis	Omega-Hydroxylase P450	At5g58860	CYP86A1 (horst)	F0SUT5C01A07GU.l.qr.1:364:1986:1	64.9	0
Cutin Biosynthesis	Omega-Hydroxylase P450	At4g00360	CYP86A2 (att1)	F0SUT5C01A07GU.l.qr.1:364:1986:1	72.3	0
Cutin Biosynthesis	Omega-Hydroxylase P450	At1g01600	CYP86A4	F0SUT5C01A07GU.l.qr.1:364:1986:1	70.5	0
Cutin Biosynthesis	Omega-Hydroxylase P450	At1g63710	CYP86A7	F0SUT5C01A07GU.l.qr.1:364:1986:1	73.7	0
Cutin Biosynthesis	Omega-Hydroxylase P450	At2g45970	CYP86A8 (lcr)	F0SUT5C01A07GU.l.qr.1:364:1986:1	75.4	0
Cutin Biosynthesis	Omega-Hydroxylase P450	At2g46960	CYP709B1	F0SUT5C01B5RU4.l.qr.1:92:1648:2	57.0	1E-134
Cutin Biosynthesis	Omega-Hydroxylase P450	At2g46950	CYP709B2	F0SUT5C01B5RU4.l.qr.1:92:1648:2	59.8	1E-175

Cutin Biosynthesis	Omega-Hydroxylase P450	At1g57750	CYP96A15 MAH1	F0SUT5C01A07GU.l.qr.1:364:1986:1	36.8	1E-81
Cutin Biosynthesis	Omega-Hydroxylase P450	At4g27710	CYP709B3	F0SUT5C01B5RU4.l.qr.1:92:1648:2	57.8	1E-166
Cutin Biosynthesis	ABC transporters	At1g51500	cer5, WBC12	F0SUT5C01AYOQH.l.qr.1:220:2259:1	63.9	0
Cutin Biosynthesis	ABC transporters	At3g21090	WBC15/WBC22	F0SUT5C01AYOQH.l.qr.1:220:2259:1	63.6	0
Cutin Biosynthesis	ABC transporters	At1g51460	WBC13	F0SUT5C01AYOQH.l.qr.1:220:2259:1	60.7	0
Cutin Biosynthesis	ABC transporters	At2g28070	WBC3	F0SUT5C01AMP6R.l.qr.1:1:965:3	67.2	1E-119
Cutin Biosynthesis	ABC transporters	At1g17840	WBC11	F0SUT5C01A8ZFZ.l.qr.1:95:1735:2	82.1	0
Cutin Biosynthesis	Various	At2g41540	G3P DH (WIN1+)	F0SUT5C01A0FPT.l.qr.1:45:1160:3	88.9	0
Cutin Biosynthesis	Various	At2g04570	Lipase (WIN1+)	F0SUT5C01AFKCV.l.qr.1:97:1143:1	64.5	1E-136
Cutin Biosynthesis	Various	At2g38110	AT (WIN1+)	F0SUT5C01BEZNH.l.qr.1:1:1575:3	59.1	1E-170
Cutin Biosynthesis	Various	At1g44170	ADH	F0SUT5C01AFK8V.l.qr.1:1:1266:1	71.6	1E-177
Cutin Biosynthesis	Various	At4g34240	ADH	F0SUT5C01AFK8V.l.qr.1:1:1266:1	64.8	1E-156
Cutin Biosynthesis	Various	At4g36250	ADH	F0SUT5C01A7K8X.l.qr.1:1:1505:3	62.0	1E-180
Cutin Biosynthesis	Bodygard	At1g64670	BDG	F0SUT5C01B0FOB.l.qr.1:158:1591:2	56.0	1E-148
Cutin Biosynthesis	Hothead	At1g72970	ACE/HTH	F0SUT5C01AJBOL.l.qr.1:84:1547:3	67.8	0
Cutin Biosynthesis	P450	At5g23190	CYP86B1	F0SUT5C01A07GU.l.qr.1:364:1986:1	46.3	1E-130
Cutin Biosynthesis	P450	At5g08250	CYP86B2	F0SUT5C01A07GU.l.qr.1:364:1986:1	47.9	1E-126
Cutin Biosynthesis	P450	At1g24540	CYP86C1	F0SUT5C01A07GU.l.qr.1:364:1986:1	37.2	7E-95
Cutin Biosynthesis	P450	At3g26125	CYP86C2	F0SUT5C01A07GU.l.qr.1:364:1986:1	36.5	1E-90
Cutin Biosynthesis	P450	At1g13140	CYP86C3	F0SUT5C01A07GU.l.qr.1:364:1986:1	37.4	1E-103
Cutin Biosynthesis	P450	At1g13150	CYP86C4	F0SUT5C01A07GU.l.qr.1:364:1986:1	40.1	1E-103
Cutin Biosynthesis	P450	At5g63450	CYP94B1	F0SUT5C01A77J4.l.qr.1:1:1569:1	47.0	1E-122
Cutin Biosynthesis	P450	At3g01900	CYP94B2	F0SUT5C01A77J4.l.qr.1:1:1569:1	42.9	1E-106
Cutin Biosynthesis	P450	At3g48520	CYP94B3	F0SUT5C01A77J4.l.qr.1:1:1569:1	48.0	1E-121
Cutin Biosynthesis	P450	At2g27690	CYP94C1	F0SUT5C01A77J4.l.qr.1:1:1569:1	45.4	1E-105
Cutin Biosynthesis	P450	At1g34540	CYP94D1	F0SUT5C01A77J4.l.qr.1:1:1569:1	44.4	1E-103
Cutin Biosynthesis	P450	At3g56630	CYP94D2	F0SUT5C01A77J4.l.qr.1:1:1569:1	43.6	1E-102
Cutin Biosynthesis	P450	At2g23180	CYP96A1	F0SUT5C01A07GU.l.qr.1:364:1986:1	38.8	8E-91
Cutin Biosynthesis	P450	At4g32170	CYP96A2	F0SUT5C01A07GU.l.qr.1:364:1986:1	35.8	1E-83
Cutin Biosynthesis	P450	At1g65340	CYP96A3	F0SUT5C01A07GU.l.qr.1:364:1986:1	37.6	8E-82
Cutin Biosynthesis	P450	At5g52320	CYP96A4	F0SUT5C01A07GU.l.qr.1:364:1986:1	37.1	5E-82
Cutin Biosynthesis	P450	At2g21910	CYP96A5	F0SUT5C01A07GU.l.qr.1:364:1986:1	35.4	7E-81
Cutin Biosynthesis	P450	At5g51900	CYP96A6P	F0SUT5C01EDB0L.l.qr.1:1:528:3	38.2	5E-14
Cutin Biosynthesis	P450	At1g47630	CYP96A7	F0SUT5C01A77J4.l.qr.1:1:1569:1	37.3	5E-63
Cutin Biosynthesis	P450	At1g47620	CYP96A8	F0SUT5C01A07GU.l.qr.1:364:1986:1	36.3	6E-81
Cutin Biosynthesis	P450	At4g39480	CYP96A9	F0SUT5C01A07GU.l.qr.1:364:1986:1	38.2	1E-81
Cutin Biosynthesis	P450	At4g39490	CYP96A10	F0SUT5C01A07GU.l.qr.1:364:1986:1	40.0	2E-92
Cutin Biosynthesis	P450	At4g39500	CYP96A11	F0SUT5C01A07GU.l.qr.1:364:1986:1	36.0	3E-76
Cutin Biosynthesis	P450	At4g39510	CYP96A12	F0SUT5C01A07GU.l.qr.1:364:1986:1	32.9	2E-69
Cutin Biosynthesis	P450	At5g02900	CYP96A13	F0SUT5C01A07GU.l.qr.1:364:1986:1	34.7	1E-70
Cutin Biosynthesis	P450	At1g66030	CYP96A14P	no hit	no hit	no hit

Cutin Biosynthesis	P450	At1g31800	CYP97A3	F0SUT5C01C57PD.l.qr.1:1:1319:3	84.7	0
Cutin Biosynthesis	P450	At4g15110	CYP97B3	F0SUT5C02F8X3N.l.qr.1:1:808:2	78.0	1E-116
Cutin Biosynthesis	P450	At3g53130	CYP97C1	F0SUT5C01D2JXV.l.qr.1:1:1035:1	83.1	1E-161
Cutin Biosynthesis	P450	At2g44890	CYP704A1	F0SUT5C02F5VXY.l.qr.1:1:1557:1	60.4	0
Cutin Biosynthesis	P450	At2g45510	CYP704A2	F0SUT5C02F5VXY.l.qr.1:1:1557:1	64.5	0
Cutin Biosynthesis	P450	At1g69500	CYP704B1	F0SUT5C02F5VXY.l.qr.1:1:1557:1	42.6	1E-110
Cutin Biosynthesis	P450	At2g45510	CYP704A2	F0SUT5C02F5VXY.l.qr.1:1:1557:1	64.5	0
Cutin Biosynthesis	P450	At1g69500	CYP704B1	F0SUT5C02F5VXY.l.qr.1:1:1557:1	42.6	1E-110
Others	others	At4g30140	CDEF1	F0SUT5C01A1KZA.l.qr.1:84:1175:3	40.4	4E-69
Others	others	At5g23940	BADH	F0SUT5C01AOIKO.l.qr.1:1:1149:1	66.8	1E-127
Others	others	At5g41040	ASFT (BADH)	F0SUT5C01A12QA.l.qr.1:1:977:1	66.4	1E-106
Others	others	At3g54010	PAS1	FFYUTAE01BV3B2.l.qr.1:1:859:2	82.0	1E-139
Others	others	At1g01280	CYP703A2	F0SUT5C01A2L2A.l.qr.1:43:1584:1	37.7	9E-92
Others	others	At1g69500	CYP704B1	F0SUT5C02F5VXY.l.qr.1:1:1557:1	42.6	1E-110
Others	others	At5g04660	CYP77A4	F0SUT5C01A1IS7.l.qr.1:1:1394:3	74.9	0
Others	others	At5g23190	CYP86B1 / ralf	F0SUT5C01A07GU.l.qr.1:364:1986:1	46.3	1E-130
Others	others	At3g10570	CYP77A6	F0SUT5C01A1IS7.l.qr.1:1:1394:3	73.9	0
Others	others	At1g01610	GPAT4	F0SUT5C01BEZNH.l.qr.1:1:1575:3	75.0	0
Others	others	At3g11430	GPAT5	F0SUT5C01EUOBU.l.qr.1:1:679:2	88.1	1E-108
Others	others	At2g38110	GPAT6	F0SUT5C01BEZNH.l.qr.1:1:1575:3	59.1	1E-170
Others	others	At4g00400	GPAT8	F0SUT5C01BEZNH.l.qr.1:1:1575:3	75.6	0
