# Supplementary material for: Bioinformatic analysis of ESTs collected by Sanger and pyrosequencing methods for a keystone forest tree species: oak
Source: BMC Genomics. 2010 Nov 23;11:650. doi: 10.1186/1471-2164-11-650 (PMC3017864; doi:10.1186/1471-2164-11-650)
Supplement: Additional file 9 — Table S5: Homology search results against MAIZEWALL database. [file 1471-2164-11-650-S9.PDF]

Table S5. Homology search results against MAIZEWALL database

Categories (listed in alphabetical order)	Putative function	Number of unigene elements in OakContigV1
Cell wall proteins	Expansin	91
	Arabinogalactan protein (AGP)	10
	Dirigent protein	24
	Extensin	37
	Glycine rich protein (GRP)	188
	Proline rich protein (PRP)	62
	Proline-rich APG-like protein	45
Cellulose synthesis	Cellulose synthase	121
	Plasma membrane protein (kobito1-2)	5
	Sucrose synthase	88
	Endo-1,3-1,4-beta-D-glucanase	162
DNA/RNA regulation and cell cycle	Actin depolymerizing factor	33
	CDK5 activator-binding protein	3
	Cyclin-dependent kinase protein kinase cdc2d	158
	DNA-binding protein	4
	DnaJ	126
	Endonuclease	11

Categories (listed in alphabetical order)	Putative function	Number of unigene elements in OakContigV1
	Histone deacetylase	9
	Histone H2A	78
	Histone H2B	58
	Histone H3	102
	Histone H4	70
	Replication protein A1	6
	Replication protein A2 (RPA2)	6
	Ribonuclease I	14
	RNA polymerase II fifth largest subunit	7
	RNA polymerase II subunit RPB10	9
	Small nuclear ribonucleoprotein	33
Flavonoids	Chalcone isomerase	8
	Chalcone synthase	46
	Dihydroflavonol reductase (DFR)	21
	Glutathion S-transferase (Bronze-2)	137
General phenylpropanoid	4-coumarate:coenzyme A ligase (4CL)	89
	Caffeic acid O-methyltransferase (COMT)	17
	Caffeoyl-CoA 3-O-methyltransferase (CCoAOMT)	51
	Cinnamyl alcohol dehydrogenase 1 (CAD1)	54
	Ferulate 5-hydroxylase (F5H)	50

Categories (listed in alphabetical order)	Putative function	Number of unigene elements in OakContigV1
	Hydroxycinnamoyl-CoA transferase (HCT)	32
	Laccase	66
	p-Coumarate 3-hydroxylase (C3H)	80
	Peroxidase	257
	Phenylalanine ammonia lyase (PAL)/Tyrosine ammonia lyase (TAL)	11
	Aldehyde deshydrogenase/Reduced epidermal fluorescence1 (REF1)/Restore fertility (RF2)	166
	Trans-cinnamate 4-hydroxylase (C4H)	34
Hormone-related	1-Aminocyclopropane-1-carboxylate synthase	3
	Abscissic acid-induced protein	13
	C-4 sterol methyl oxidase	9
	Gibberellin-regulated protein GAST1/GASA like	62
	GTP-binding protein SAR1A	85
	Plasma membrane protein (pin1)	9
	Serine/Threonine kinase (pinoid)	169
	Sterol methyltransferase	22
	Dehydration-induced protein RD22	2
	Wol histidine kinase	25
Hydroxylation and O-methylation enzymes	Cytochrome P450	215
	O-methyltransferase (OMT)	60

Categories (listed in alphabetical order)	Putative function	Number of unigene elements in OakContigV1
	S-adenosyl-L-methionine:salicylic acid carboxyl methyltransferase	17
	S-adenosyl-methionine synthetase 3 (SAMS)	82
Lignin/lignan	Pinoresinol reductase	80
	Secoisolariciresinol dehydrogenase	130
	Cinnamoyl-coA reductase (CCR)	46
	Cinnamyl alcohol dehydrogenase 2 (CAD2)	112
Non cellulosic polysaccharide biosynthesis	Xylosyltransferase	15
	Putative glycosyltransferase (quasimodo)	54
	Callose synthase	67
	Cellulose synthase-like (Csl)	11
	Fucosyltransferase	3
	Galactosyltransferase	45
	Glucosyl transferase	285
	1,4-alpha-glucan branching enzyme	24
Nucleotide sugar synthesis, conversion and transport	GDP mannose pyrophosphorylase	20
	GDP mannose transporter	32
	GDP-mannose 4,6-dehydratase (mur1)	5
	Xyloglucan galactosyltransferase (mur3)	5

Categories (listed in alphabetical order)	Putative function	Number of unigene elements in OakContigV1
	UDP-D-galactose 4-epimerase (mur4)	12
	Putative fructokinase II	28
	UDP-glucose 6-dehydrogenase	42
	UDP-glucuronic acid decarboxylase	74
	Xylose isomerase	14
	UDP-D-glucose 4-epimerase	12
Polysaccharide modifying enzymes	Alpha-L-arabinofuranosidase	3
	Alpha-mannosidase	22
	Cell wall invertase	20
	Chitinase	10
	Chitinase like	112
	Exoglucanase	45
	Galactosidase	34
	Glucosidase	186
	Mannosyl-oligosaccharide mannosidase	11
	Pectate lyase	39
	Pectin methyl esterase	108
	Pectinesterase	36
	Polygalacturonase	56

Categories (listed in alphabetical order)	Putative function	Number of unigene elements in OakContigV1
	Xylanase	4
	Xyloglucan endotransglycosylase/hydrolase (XTH)	150
	Xylosidase	17
Protein processing	Cucumisin	77
	Cysteine proteinase	151
	Putative signal peptidase	6
	Ubiquitin/ribosomal protein CEP52	413
	Ubiquitin-conjugating enzyme E2	168
Protein synthesis	Ribosomal protein L28	17
	60S Ribosomal proteins	264
	40S Ribosomal proteins	186
Putative transporters and trafficking	ABC transporter	71
	ADP/ATP carrier protein	141
	Dolichyl-di-phosphooligosaccharide-protein glycotransferase	18
	Emb30	5
	MtN21 nodulin-like protein	74
	Nucleoside transporter	7
	Oligopeptide transporter	100
	SEC14-like protein	26

Categories (listed in alphabetical order)	Putative function	Number of unigene elements in OakContigV1
	Syntaxin-related protein KNOLLE	22
	Two component response regulator-like protein	18
Shikimate pathway	Chorismate mutase	12
	Prephenate dehydratase	13
Signaling	Annexin	32
	Calmodulin	123
	Calreticulin	46
	Preprophytosulfokine	1
	Wall-associated kinase	1782
Transcription factors	Zinc finger protein	18
	Transcription regulatory protein	1
	SHP1 MADS-BOX	58
	MYB transcription factor	132
	Transcription factor (Monopteros)	51
	Lim protein	32
	HD-ZIP protein (ifl1)	8
	Histone promoter-binding protein HBP-1A(C14) transcription factor	0
	HD domain protein	2
	bZIP transcription factor	12
	ATHB-8 HD-zip protein	6

Categories (listed in alphabetical order)	Putative function	Number of unigene elements in OakContigV1
	MYB-like transcription factor (APL)	54
Unknown genes	Unknown gene (close to DV017507)	15
	Unknown gene (close to DV017148)	11
	Unknown gene (close to DV017342)	11
	Unknown gene (close to DV017208)	1
	Unknown gene (close to DV017393)	3
	Unknown gene (close to DV017352)	1
	Unknown gene (close to DV017580)	7
	Unknown gene (close to DV017236)	3
	Unknown gene (close to DV017343)	1
	Unknown gene (close to DV017588)	10
	Unknown gene (close to DV017378)	2
	Unknown gene (close to DV017478)	4
Miscellaneous	Argonaute	53
	3-beta-hydroxysteroid-delta(8), delta(7)-isomerase	3
	Alcohol dehydrogenase	3
	Benzoquinone reductase-like	25
	Integral membrane protein (cov-1)	15
	Cytochrome b5	38
	Exportin1 protein (XPO1)	5

Categories (listed in alphabetical order)	Putative function	Number of unigene elements in OakContigV1
	Plasmodesmal associated protein SE-WAP41	44
	GTP cyclohydrolase I	1
	Katanin like	108
	Kinetochore (SKP1)-like protein	30
	Late-embryogenesis protein lea5 like	0
	Mago Nashi protein homolog	6
	NADPH-ferrihemoprotein reductase	12
	Non-symbiotic hemoglobin protein	5
	Nuclear transport factor 2 (NTF2)	8
	Nucleolar protein	8
	Pescadillo-like protein	3
	PGPD14 protein	32
	Protein kinase CK2	20
	Protein phosphatase 2C	134
	Proteinase inhibitor/gamma-thionin	20
	Putative cyclin-dependent kinase regulatory subunit	9
	Sesquiterpene cyclase I	44
	Stress-induced protein sti1	29
	Substilisin/chymotrypsin-like inhibitor	0
	WD repeat protein	3

Categories (listed in alphabetical order)	Putative function	Number of unigene elements in OakContigV1
	Lipid transfer protein/tracheary element differentiation protein 4 (TED4)	3
